# Modifiable pre-operative physiological predictors of length of stay following shoulder arthroplasty: a systematic review

**DOI:** 10.1016/j.xrrt.2026.100863

**Published:** 2026-08-24

**Authors:** Hussayn Shinwari, Bilal Ceesay, Hamzah Chaudhry, Hanan Taimur Shinwari, Wisal Khan, Usman Tahir, Arnau Sheikh, Jawwad Mihran Haider, Danielle Page, Abith Ganesh Kamath, Saran Singh Gill, Kapil Sugand

**Affiliations:** a Worcestershire Acute Hospitals NHS Trust, Worcestershire Royal Hospital, Worcester, UK; b Faculty of Medicine, St George’s University of London, London, UK; c Faculty of Medicine, University of Sheffield, Sheffield, UK; d lnstitute of Biotechnology and Genetic Engineering (IBGE), The University of Agriculture Peshawar, Pakistan; e Barts Health NHS Trust, The Royal London Hospital, London, UK; f St George’s University Hospitals NHS Foundation Trust, St George’s Hospital, London, UK; g Faculty of Medicine, Imperial College London, London, UK

**Keywords:** Shoulder arthroplasty, Hospital length of stay, Pre-operative risk factors, Frailty, Anemia, Malnutrition, Diabetes mellitus, Opioid use

## Abstract

**Background:**

Shoulder arthroplasty volumes have increased markedly, intensifying pressure on hospital resources. Prolonged hospital length of stay (LOS) elevates costs, complications, and societal burden. Identifying modifiable pre-operative predictors of LOS may enable targeted optimization to improve outcomes and efficiency.

**Methods:**

A systematic search of PubMed, Embase, Medline, Scopus, and Web of Science was conducted through July 20, 2025, following Preferred Reporting Items for Systematic Reviews and Meta-Analyses 2020 and Synthesis Without Meta-analysis guidelines. Studies including adults undergoing shoulder arthroplasty, reporting patient- or procedure-related factors associated with LOS, were included. Due to heterogeneity, qualitative synthesis was performed.

**Results:**

Sixty-four studies (n = 2,435,166) were included. Prolonged LOS was consistently associated with anemia (7 studies, 1.8-5.0 days depending on severity), malnutrition or nutritional risk (2 studies, 2.2-3.7 days), hypoalbuminemia (3 studies, 2.3-3.5 days), insulin-dependent or complicated diabetes (9 studies, 0.5-0.7-day increase), higher American Society of Anesthesiologists grade (7 studies, 2.6-4.3 days), frailty or functional indices (3 studies, graded increases), cumulative comorbidity burden (≥10 studies), and pre-operative opioid use (7 studies, 1.4-2.3 days). Dehydration (2 studies), electrolyte disturbances (3 studies), and mental health disorders particularly depression (7 studies, 2.0-3.0 days) were also associated with longer LOS. Morbid obesity (body mass index ≥40 kg/m^2^) modestly increased LOS (mean difference 0.3-0.4 days), whereas general obesity (body mass index 30-40) showed inconsistent associations across 13 studies. Global physiological measures (American Society of Anesthesiologists grade, frailty, comorbidity burden) outperformed individual comorbidities in predicting LOS.

**Conclusions:**

Physiological reserve, metabolic factors, and mental health are key modifiable determinants of LOS after shoulder arthroplasty. Pre-operative optimization targeting anemia, nutrition, glycemic control, and mental health may reduce hospital LOS and facilitate short-stay or same-day pathways. These findings provide an evidence-based framework for perioperative planning to improve patient outcomes and resource utilization.

Shoulder arthroplasty is indicated for glenohumeral pathology and proximal humeral fractures causing pain and functional limitation. In the United States, annual shoulder arthroplasty volume increased from approximately 12,000 procedures in 2000 to over 110,000 by 2019, with continued growth projected.^12^ International registry data show similar trends, with annual growth rates of 6%-15% in Europe and Asia over the last decade (2011-2019).^76^ This increasing procedural volume places substantial pressure on perioperative resources, hospital capacity, and surgeon workload.

Hospital length of stay (LOS) after shoulder arthroplasty is a key determinant of health-care efficiency, cost, and patient outcomes.^20^ Prolonged LOS is associated with increased complications, delayed access to care, and greater health-care and societal burden.^20^^,^^32^^,^^42^ Extended LOS also negatively impacts patient quality of life and recovery trajectories and contributes to societal costs through lost productivity and caregiver burden.^15^^,^^34^ Identifying modifiable pre-operative predictors of LOS is therefore critical for optimizing outcomes and supporting short-stay or same-day arthroplasty pathways.

This systematic review aims to identify and synthesize modifiable pre-operative physiological predictors of LOS following shoulder arthroplasty, offering actionable insights to inform clinical optimization strategies and enhance health-care efficiency. We hypothesized that modifiable pre-operative physiological, metabolic, and psychosocial factors are associated with prolonged LOS following shoulder arthroplasty, and that these factors can be synthesized into clinically actionable domains to inform perioperative optimization strategies.

## Materials and methods

### Adherence to guidelines

The systematic review was conducted in line with the Preferred Reporting Items for Systematic Reviews and Meta-Analyses 2020 guidelines.^10^ The completed Preferred Reporting Items for Systematic Reviews and Meta-Analyses flowchart is shown in Figure 1. Analysis was conducted in line with Synthesis Without Meta-analysis guidelines.^11^

**Figure 1:**
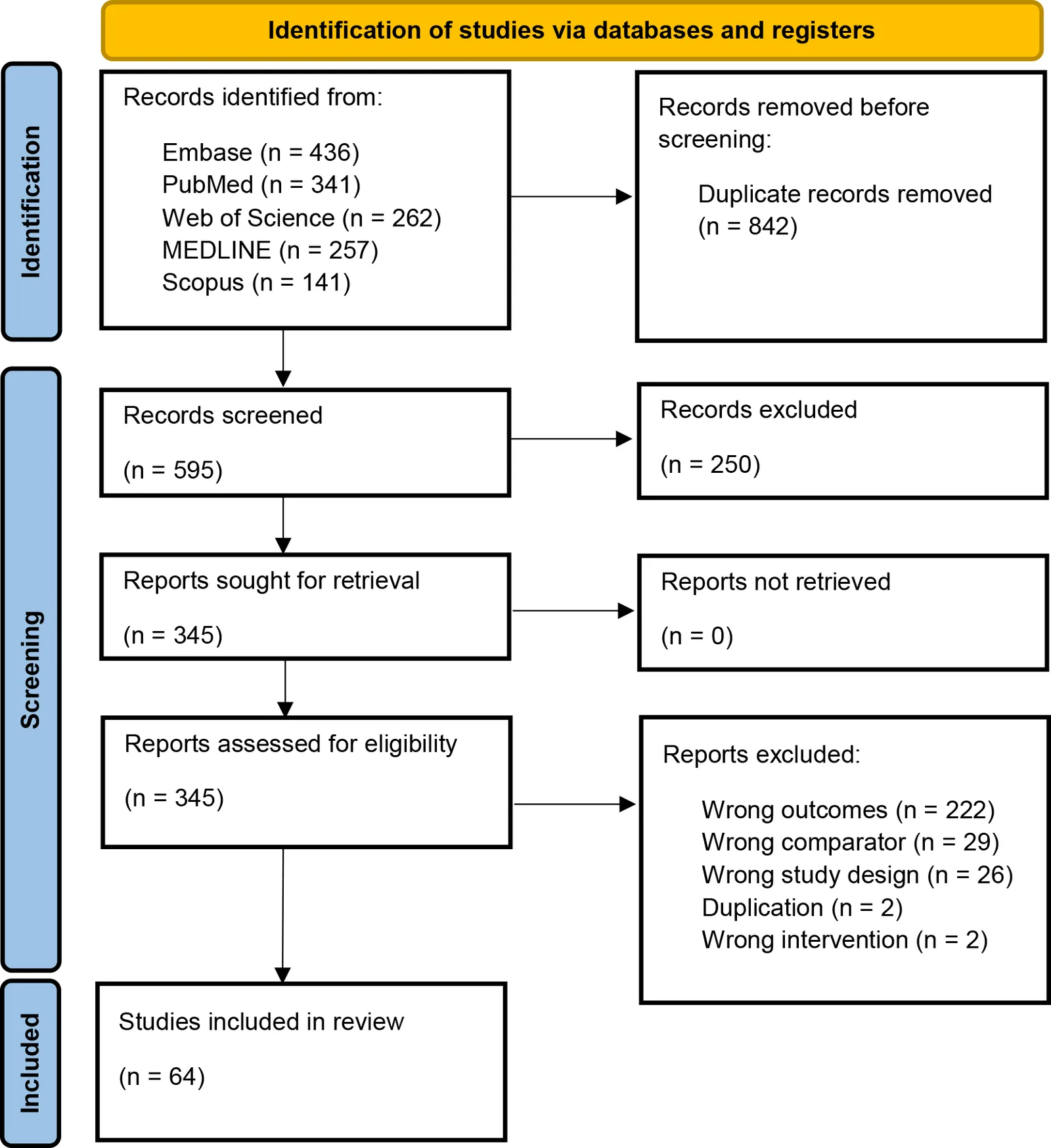
PRISMA flow diagram. *PRISMA*, Preferred Reporting Items for Systematic Reviews and Meta-Analyses.

### International Prospective Register of Systematic Reviews registration

This systematic review is registered with International Prospective Register of Systematic Reviews (PROSPERO) (registration number: CRD420261284138).

### Electronic databases

A multidatabase search was performed from conception to July 20, 2025, encompassing PubMed, Embase, Medline, Scopus, and Web of Science. Our full search string can be found in Supplementary Table S1.

### Eligibility criteria

Eligible studies included adult patients (≥18 years) undergoing shoulder arthroplasty that evaluated patient- or procedure-related factors associated with LOS, prolonged LOS, or risk of extended hospitalization. Observational studies, including retrospective and prospective cohort studies, case-control studies, and registry/database analyses, were included. Studies were excluded if they involved pediatric populations, non-arthroplasty procedures, interventions unrelated to LOS, or lacked LOS data or a comparator group. Only full-text articles published in English were considered. Supplementary Table S2 outlines the Population, Intervention, Comparison, and Outcomes criteria.

### Screening and data extraction

Studies were imported into Covidence, where duplicates were removed, and titles/abstracts screened independently by three reviewers (H.S., B.C., H.C.) following Cochrane recommendations. Full-text articles were reviewed for eligibility based on the Population, Intervention, Comparison, and Outcomes framework. Data extraction was performed independently by three reviewers (H.S., B.C., H.C.). Discrepancies were resolved by discussion, with final arbitration by the senior author (K.S.). Data were extracted using an Excel (Microsoft Inc., Washington, USA) spreadsheet including study characteristics, study design, patient demographics, location, publication year, sample size, and outcomes assessed.

### Risk of bias

Two independent reviewers (H.C. and B.C.) assessed the risk of bias using Risk Of Bias In Non-randomized Studies – of Interventions (ROBIN-I) for non-randomized studies as shown in Figure 2.^14^ Disagreements were resolved through consensus, with a third reviewer (H.S.) providing arbitration when necessary. The assessment focused on confounding, missing data, and selective reporting.

**Figure 2:**
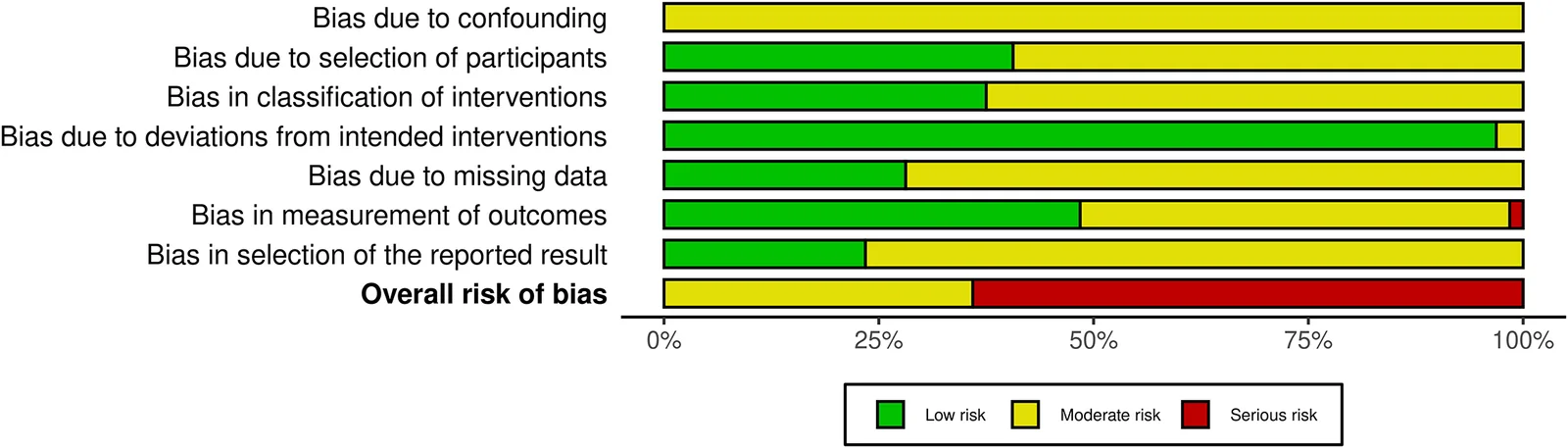
ROBINS-I risk of bias assessment. ROBINS-I, Risk Of Bias In Non-randomized Studies – of Interventions.

### Data analysis, qualitative synthesis, and reporting

Due to substantial heterogeneity across studies in arthroplasty type (anatomic vs. reverse), surgical indications, perioperative protocols, and definitions of LOS (continuous vs. dichotomous), quantitative meta-analysis was not feasible. Instead, a qualitative synthesis was conducted as per Synthesis Without Meta-analysis guidance.^11^

## Results

### Study design

A total of 988 articles were initially identified (Fig. 1). After removal of duplicates and detailed screening against predefined inclusion and exclusion criteria, 64 studies involving a total of 2,435,166 patients were deemed eligible and included in the final analysis.^2^, ^21^, ^22^, ^23^, ^3^, ^4^, ^5^^,^^7^^,^^8^^,^^10^^,^^11^^,^^16^, ^17^, ^18^, ^19^^,^^21^, ^22^, ^23^^,^^25^, ^26^, ^27^, ^28^, ^29^, ^30^, ^31^^,^^35^, ^36^, ^37^, ^38^, ^39^, ^40^^,^^42^, ^43^, ^44^, ^45^^,^^47^, ^48^, ^49^, ^50^, ^51^, ^52^, ^53^, ^54^^,^^56^, ^57^, ^58^, ^59^, ^60^, ^61^, ^62^, ^63^, ^64^, ^65^, ^66^, ^67^^,^^69^^,^^70^^,^^72^, ^73^, ^74^, ^75^^,^^78^^,^^79^^,^^81^, ^82^, ^83^, ^84^

### Study characteristics

Patient characteristics varied across studies, with mean ages ranging from 60 to 78 years and sample sizes from 29 to over 422,000 patients (Table I and II). The studies were predominantly conducted in the United States (n = 62), with smaller contributions from the United Kingdom (n = 1) and Spain (n = 1). The majority of studies were retrospective cohort studies.

**Table 1:** Patient demographics.

Author (yr)	Title	Journal	Study design	Country or region	Data source	Sample size (total and for each sub-group (if applicable)	Age (mean ± SD or median (IQR))	% Male	BMI (mean ± SD)	Comorbidity indices	Indication for TSA	Type of TSA performed
Liu et al 2025^50^	Dehydration independently predicts prolonged hospital stay following aseptic revision total shoulder arthroplasty.	European Journal of Orthopaedic Surgery and Traumatology	Retrospective cohort study	United States	American College of Surgeons National Surgical Quality Improvement Program (ACS-NSQIP) database (2015-2022)	1,372	Age reported categorically - overall cohort: 0.8% of normally hydrated and 0.4% of dehydrated patients were 18-39 yr; 16.5% vs. 8.0% were 40-59 yr; 72.5% vs. 72.9% were 60-79 yr; and 10.2% vs. 18.7% were ≥80 yr	Overall cohort: 62.9% male. In the normal hydration group, 65.6% were male vs. 49.3% in the dehydration group	Mean BMI not reported but BMI presented categorically - <18.5 kg/m^2^ (0.1% normal vs. 0% dehydrated), 18.5-24.9 kg/m^2^ (14.1% vs. 16.4%), 25-29.9 kg/m^2^ (30.9% vs. 32.4%), and ≥30 kg/m^2^ (54.9% vs. 51.1%)	ASA classification ≤2 in 34.7% of normal hydration and 27.1% of dehydrated patients; ASA ≥3 in 65.3% vs. 72.9% Comorbidities: CHF 1.9% vs. 3.6%, hypertension 70.3% vs. 76.9%, COPD 6.6% vs. 10.7%, bleeding disorder 3.7% vs. 4.4%, disseminated cancer 0.3% vs. 0% Functional status was independent in 96.7% vs. 97.8%	Aseptic (non-infectious) revision TSA; cases performed for infection were excluded	Revision total shoulder arthroplasty
Nazzal et al 2025^67^	Predictors of failing same-day discharge after shoulder arthroplasty: developing a model to improve outcomes and reduce health care cost.	Journal of Shoulder and Elbow Surgery	Retrospective case-control study	United States	Single-center institutional database (consecutive shoulder arthroplasty cohort)	333	69.9 ± 7.9 yr overall. Achieved SDD had a mean age of 69.5 ± 7.7 vs. 71.1 ± 8.2 yr in patients who failed SDD.	46.9% male overall; 50.2% male in the SDD group vs. 38.0% male in the failure group.	Mean BMI 30.9 ± 6.4 kg/m^2^; SDD group 30.5 ± 6.4 vs. failure group 32.0 ± 6.3.	Mean Charlson Comorbidity Index (CCI) 3.4 ± 1.6 overall (3.33 ± 1.6 for SDD vs. 3.67 ± 1.7 for failure)	Primary glenohumeral osteoarthritis or rotator cuff arthropathy. Patients with fractures, revision arthroplasty, or age <45 yr were excluded.	Anatomic TSA, reverse TSA
Areti et al 2025^4^	Impact of morbid obesity on post-operative outcomes in reverse total shoulder arthroplasty: A national inpatient sample analysis.	Journal of Orthopaedics	Retrospective cohort study	United States	National Inpatient Sample (NIS), Healthcare Cost and Utilization Project (HCUP)	59,925	Mean age at admission: morbidly obese 67.74 ± 7.674 yr vs. 71.67 ± 8.642 yr in the control group (difference −3.93 yr, *P* < .001). Age categories showed a greater proportion of younger patients in the morbidly obese cohort (<60 yr: 14.15% vs. 8.32% control; 60-69 yr: 42.55% vs. 29.84%; *P* < .001).	28.40% in morbidly obese vs. 40.44% male control group.		Diabetes and tobacco-related disorders. Diabetes without complications occurred in 22.04% of morbidly obese vs. 13.78% controls; diabetes with complications occurred in 0.39% vs. 0.18%. Tobacco-related diagnoses were less common in the morbidly obese group (11.26% vs. 16.51%, *P* < .001).	Rotator cuff arthropathy, fractures, osteoarthritis and revision arthroplasty	Reverse total shoulder arthroplasty arthroplasty only
Mayfield et al 2025^60^	Perioperative opioid use in total shoulder arthroplasty is associated with dose-dependent risk of major surgical and medical complications.	Journal of Shoulder and Elbow Surgery	Retrospective cohort study	United States	Premier Healthcare Database (PHD)	140,251	1.70 ± 8.51 yr in the lowest quintile, 70.66 ± 8.59 yr, 70.00 ± 8.85 yr, 69.02 ± 9.02 yr and 67.24 ± 9.48 yr in the highest quintile.	45.06% in the lowest quintile vs. 40.62% in the highest quintile		Congestive heart failure, chronic pulmonary disease, complicated diabetes, obesity, fluid electrolyte imbalance, and depression	Primary elective anatomic or reverse total shoulder arthroplasty	Anatomic TSA, reverse TSA
Liu et al 2024^54^	Severe malnutrition predicts early post-operative complications in aseptic revision total shoulder arthroplasty.	Shoulder and Elbow	Retrospective cohort study	United States	ACS-NSQIP database	678	Age reported by categories: 65-74 yr (67.6% normal, 60.0% moderate, 52.3% severe); 75-84 yr (29.5% normal, 32.6% moderate, 36.9% severe); ≥85 yr (2.9% normal, 7.4% moderate, 10.8% severe)	Based on Geriatric Nutritional Risk Index (GNRI): normal GNRI 45.6% male, moderate malnutrition 47.4% male, severe malnutrition 40.0% male		ASA classification ≥3: 68.3% normal nutrition, 78.9% moderate malnutrition and 83.1% severe malnutrition, Comorbidities recorded included congestive heart failure (CHF), diabetes, hypertension, chronic obstructive pulmonary disease (COPD), bleeding disorders and disseminated cancer; COPD was more prevalent in the moderate malnutrition group (13.7% vs. 7.1% normal)	Revision total shoulder arthroplasty for non-infectious etiologies	Revision total shoulder arthroplasty
Mayfield et al 2025^61^	Tranexamic acid use in total shoulder arthroplasty continues to increase and is safe in high-risk patients.	Journal of Shoulder and Elbow Surgery	Retrospective cohort study	United States	Premier Healthcare Database (PHD)	86,356	Mean age was 70.3 ± 8.4 yr (TXA^+^) vs. 70.5 ± 8.6 yr (TXA^−^). High-risk cohorts had similar mean ages (71.5 vs. 71.6 yr).	45.3% of the TXA^+^ group and 44.9% of the TXA^−^ group were male. In the high-risk sub-group, 39.7% (TXA^+^) and 38.9% (TXA^−^) were male.		In the unmatched cohorts, TXA^+^ patients had lower rates of congestive heart failure (4.9% vs. 6.1%), pulmonary circulation disorders (0.9% vs. 1.1%), peripheral vascular disease (3.1% vs. 3.8%), complicated hypertension (10.8% vs. 11.8%), renal failure (8.4% vs. 9.4%) and history of VTE (4.5% vs. 6.5%). Rates of chronic pulmonary disease, diabetes, hypothyroidism, obesity, depression and other comorbidities were similar between groups. After matching, comorbidity differences were eliminated, with similar prevalence of congestive heart failure (∼5.1%), hypertension (∼60%), chronic pulmonary disease (∼19.5%) and obesity (∼21.7%) in both cohorts.	Primary elective Total shoulder arthroplasty	Anatomic and Reverse total shoulder arthroplasty
Liu et al 2024^49^	The association of hyponatremia and early post-operative complications in aseptic revision total shoulder arthroplasty.	European Journal of Orthopaedic Surgery and Traumatology	Retrospective cohort study	United States	ACS-NSQIP database	1,927	Age was categorized; 60-79 yr accounted for 71.7% of eunatremic and 71.3% of hyponatremic patients. Younger (<60 yr) and ≥80 yr groups comprised the remainder	Sex distribution was similar: eunatremia 47.7% male vs. hyponatremia 46.3% male	BMI categories: eunatremia—53.9% obese (≥30 kg/m^2^), 30.6% overweight (25-29.9 kg/m^2^), 15.1% normal weight (18.5-24.9 kg/m^2^) and 0.4% underweight; hyponatremia—46.3% obese, 26.5% overweight, 26.5% normal weight and 0.7% underweight. Hyponatremia was significantly associated with BMI <24.9 kg/m^2^ (*P* = .004)	Functional status prior to surgery (dependent vs. independent), ASA classification, smoking, steroid use and comorbidities such as congestive heart failure, hypertension, COPD, bleeding disorders and disseminated cancer were recorded	Revision total shoulder arthroplasty for aseptic indications	Anatomic and Reverse total shoulder arthroplasty
Liu et al 2024^51^	Hypoalbuminemia predicts early post-operative complications following noninfectious revision total shoulder arthroplasty.	European Journal of Orthopaedic Surgery and Traumatology	Retrospective cohort study	United States	ACS-NSQIP database	839		Normal-albumin group, 48.3% were male (362/750); in the hypoalbuminemia group 37.1% were male (33/89)	BMI was categorized: in the normal-albumin cohort, 16.9% were normal weight (18.5-24.9 kg/m^2^), 29.3% overweight (25-29.9 kg/m^2^) and 53.3% obese (≥30 kg/m^2^); in the hypoalbuminemia group 13.5% were normal weight, 73.0% overweight and 13.5% obese	ASA class and comorbidities were recorded. Hypoalbuminemia was associated with dependent functional status (10.1% vs. 2.7%; *P* < .001) and ASA class ≥3 (78.7% vs. 63.5%; *P* = .005). Diabetes was more common in the normal-albumin group (19.1% vs. 5.6%; *P* = .007)	Aseptic revision total shoulder arthroplasty	Revision total shoulder arthroplasty
Liu et al 2024^53^	Elevated alkaline phosphatase independently predicts early post-operative complications in noninfectious revision total shoulder arthroplasty.	European Journal of Orthopaedic Surgery and Traumatology	Retrospective cohort study	United States	ACS-NSQIP database	936	<40 yr 1.0% vs. 3.7% (normal vs. elevated); 40-59 yr 18.0% vs. 20.4%; 60-79 yr 71.1% vs. 64.8%; ≥80 yr 9.9% vs. 11.1%.	Normal group: 49.4% male elevated group: 35.2% male.		Normal group vs. elevated group: ASA class ≥3 in 65.1% vs. 88.9% dependent functional status: (11.1% vs. 3.3%) diabetes (31.5% vs. 19.8%) and bleeding disorders (9.3% vs. 3.1%)	Revision TSA for aseptic etiologies	Revision total shoulder arthroplasty
Liu et al 2024^52^	The geriatric nutritional risk index as a strong predictor of adverse outcomes following total shoulder arthroplasty.	European Journal of Orthopaedic Surgery and Traumatology	Retrospective cohort study	United States	ACS-NSQIP database	11,411	65-74 yr (29.1% of normal, 23.4% moderate, 21.9% severe) 75-84 yr (54.1% normal, 52.8% moderate, 48.1% severe) ≥85 yr (16.9% normal, 23.9% moderate, 30.0% severe)	Normal GNRI 42.0% male, moderate 36.6% male, severe 28.3% male	BMI not provided as a continuous measure	Functional status: 2.3% of the normal, 3.4% of the moderate and 10.4% of the severe malnutrition group ASA classification ≥3 occurred in 61.6%, 73.1% and 80.7% of normal, moderate, and severe group		Anatomic and reverse TSA
Fassler et al 2024^22^	Components of metabolic syndrome as significant risk factors for post-operative complications following total shoulder arthroplasty: hypertension, diabetes, and obesity.	Journal of Shoulder and Elbow Surgery	Retrospective cohort study	United States	ACS-NSQIP database	26,613	<65 yr 27.7% of no-MetS vs. 25.7% of MetS; 65-74 yr 40.9% vs. 49.0% ≥75 yr 30.8% vs. 25.1%. Patients with MetS were more likely to be aged 65-74 and less likely to be ≥75 y	Males 44.6% vs. 42.2% no-MetS vs. MetS		MetS vs. control ASA ≥3 was present in 85.5% vs. 54.2% dependent functional status (3.4% vs. 1.9%) Dyspnea on exertion or at rest, COPD (8.5% vs. 6.4%) Congestive heart failure (1.4% vs. 0.6%) Open wound/wound infection (0.6% vs. 0.3%) Bleeding disorders (3.3% vs. 2.4%)	Primary total shoulder arthroplasty for non-trauma indications	Anatomic and reverse TSA
Burns et al 2024^11^	Chronic kidney disease increases cost of care and readmission risk after shoulder arthroplasty.	journal shoulder elbow surgery	Retrospective cohort study	United States	Regional health system electronic medical record database (Epic; 7-hospital system)	514	CKD: 73.2 ± 8.9 yr Non-CKD: 68.1 ± 9.4 yr (*P* < .001)	CKD: 32% male Non-CKD: 53.5% male	CKD: 31.5 ± 7.2 Non-CKD: 31.7 ± 6.5 (NS)	Number of comorbidities: CKD 5.9 ± 2.9 vs. Non-CKD 5.0 ± 3.1 (*P* < .001) Specific comorbidities: CKD patients more likely to have diabetes (36.8% vs. 20.1%, *P* < .001), cardiac disease (40.8% vs. 30.3%, *P* = .03), anemia (41.9% vs. 18.5%, *P* < .001), dialysis (5.6% vs. 0, *P* < .001).	Elective primary TSA	Anatomic and Reverse TSA
Brandner et al 2024^8^	Impact of opioid dependence on outcomes following total shoulder arthroplasty.	Journal of Shoulder and Elbow Surgery	Retrospective cohort study	United States	National Readmissions Database (NRD), HCUP	58,975					Primary anatomic and reverse TSA	Anatomic and Reverse TSA
Gruson et al 2023^29^	Does morbid obesity (BMI ≥40 kg/m^2^) impact operative time, blood loss, length of stay, or complications following anatomic total shoulder arthroplasty?	Archives of bone and joint surgery	Retrospective cohort study	United States	Single-institution retrospective cohort	130	Median ages: non-obese 64.0 yr (IQR 56-71), obese 61.5 yr (IQR 55-69), morbidly obese 62.0 yr (IQR 56-67)	42% (55/130)	Median BMI: non-obese 26.8 kg/m^2^ (IQR 24.9-28.2), obese 33.9 kg/m^2^ (IQR 32.4-36.5), morbidly obese 42.7 kg/m^2^ (IQR 41.3-45.4). Overall mean BMI 33.2 ± 6.8 kg/m^2^	Age-adjusted Charlson Comorbidity Index (ACCI); medians: 3.0 (IQR 2.0-4.0) non-obese, 2.5 (IQR 1.5-4.0) obese, 2.0 (IQR 2.0-3.0) morbidly obese	Elective primary aTSA for primary osteoarthritis	Anatomic total shoulder arthroplasty
Papalia et al 2023^69^	Inpatient charges, complication, and revision rates for shoulder arthroplasty in Parkinson disease: a regional database study.	JSES International	Retrospective cohort study	United States	Statewide all-payer administrative database	39,011	Non-PD 68.6 ± 10.4 yr vs. PD 72.3 ± 8.0 yr	43%		Elixhauser Comorbidity Index	Indication by ICD-9/10 codes	Anatomic TSA, reverse TSA, and hemiarthroplasty
Diamond et al 2023^18^	How does depressive disorder impact outcomes in patients with glenohumeral osteoarthritis undergoing primary reverse shoulder arthroplasty?	JSES International	Retrospective case-control study	United States	National claims database	24,326		47.38% males DD vs. 47.36% no DD		Chronic obstructive pulmonary disease, congestive heart failure, diabetes, hyperlipidemia, hypertension, obesity.	Glenohumeral osteoarthritis or rotator cuff tear	Reverse shoulder arthroplasty
Polisetty et al 2023^72^	Iron deficiency anemia is associated with increased medical and implant-related complications and length of stay for patients undergoing total shoulder arthroplasty.	JSES International	Retrospective cohort study	usa	Medicare database	106,134	73 yr for both cohorts (age categories: <64 yr 10.25%, 65-69 yr 22.04%, 70-74 yr 24.85%, 75-79 yr 23.28%, 80-84 yr 14.33%, ≥85 yr 5.26%)	34.74% male		Elixhauser Comorbidities Index used. Matching variables included diabetes mellitus, hyperlipidemia, hypertension, obesity and tobacco use	Primary total shoulder arthroplasty	Primary total shoulder arthroplasty
Puzzitiello et al 2022^75^	Patients with limited health literacy have worse pre-operative function and pain control and experience prolonged hospitalizations following shoulder arthroplasty.	Journal Of Shoulder And Elbow Surgery	Retrospective cohort study	United States	Prospectively maintained institutional registry (OBERD database)	230	Mean age: HHL 67.9 ± 7.7 yr vs. LHL 69.0 ± 9.6 yr (*P* = .36).	HHL 50% vs. LHL 51.7% (*P* = .82)	HHL 30.8 ± 6.4 kg/m^2^ vs. LHL 31.6 ± 6.9 kg/m^2^ (*P* = .42)	ASA physical-status classification (ASA >2) and Area Deprivation Index (ADI)	Osteoarthritis or rotator cuff arthropathy	Reverse TSA and anatomic TSA
Burns et al 2022^10^	Modifiable risk factors increase length of stay and 90-d cost of care after shoulder arthroplasty.	Journal Of Shoulder And Elbow Surgery	Retrospective cohort study	United States	Regional health system electronic medical record database (Epic; 7-hospital system)	1,317				Modifiable risk factors (MRFs) defined as anemia (hemoglobin <10 g/dL), malnutrition (albumin <3.4 g/dL), obesity (BMI >40 kg/m^2^), uncontrolled diabetes (random glucose >180 mg/dL or HbA1c > 8%), tobacco use, and opioid use.	Acute fracture	
Doan et al 2021^19^	Increasing severity of anemia is associated with poorer 30-d outcomes for total shoulder arthroplasty.	JSES International	Retrospective cohort study	United States	ACS-NSQIP database	15,185	Normal HCT: 68.6 ± 9.4 yr; Mild anemia: 71.4 ± 9.1 yr; Moderate/severe anemia: 72.5 ± 10.3 yr.	50.8%, 23.1% and 22.6% respectively.	No anemia: 31.4 ± 6.7 kg/m^2^; Mild anemia: 31.1 ± 7.3 kg/m^2^; Moderate/severe anemia: 29.8 ± 7.3 kg/m^2^.	ASA physical-status classification. CHF, COPD, cancer, diabetes, dialysis, dyspnea, hypertension, renal failure, sepsis, smoking, steroid use, weight loss	Various diagnoses	Anatomic and reverse TSA
Flamant et al 2021^23^	Malnutrition in elective shoulder arthroplasty: a multi-institutional retrospective study of pre-operative albumin and adverse outcomes.	JSES	Retrospective cohort study	United States	Two tertiary institutional centers (multi-institutional retrospective cohort)	492	65.7 yr for those with albumin <3.5 g/dL vs. 66.8 yr for those with albumin ≥3.5 g/dL	42.5% of patients with low albumin were male vs. 49.6% with normal albumin	mean BMI 31.3 kg/m^2^ in malnourished patients vs. 30.4 kg/m^2^ in normoalbuminemic patients	American Society of Anesthesiologists (ASA) physical-status classification and selected Elixhauser comorbidities (congestive heart failure, pulmonary disease, diabetes, renal failure, liver disease, smoking)	Elective primary or revision total shoulder arthroplasty	Anatomic total shoulder arthroplasty (TSA) and reverse TSA (rTSA)
Wang et al 2021^82^	Increased severity of anemia is associated with post-operative complications following primary total shoulder arthroplasty.	JSES	Retrospective cohort study	United States	ACS-NSQIP database	13,921	Non-anemic: 69.12 ± 9.38 yr; mild anemia: 71.51 ± 9.31 yr; moderate/severe anemia: 72.98 ± 9.98 yr.	43.6% in non-anemic, 49.3% in mild anemia and 24.0% in moderate/severe anemia.	Non-anemic: 31.51 ± 6.84 kg/m^2^; mild anemia: 30.78 ± 6.87 kg/m^2^; moderate/severe anemia: 30.02 ± 6.92 kg/m^2^.	ASA physical-status classification (grades I-IV) and individual comorbidities (COPD, CHF, hypertension, dialysis, renal failure, weight loss, steroid use, bleeding disorder, diabetes, dyspnea)	Primary TSA for Osteoarthiritis	Anatomic TSA, reverse TSA and total shoulder resurfacing
Lunati et al 2021^57^	Pre-operative depression is a risk factor for complication and increased health care utilization following total shoulder arthroplasty.	JSES	Retrospective cohort study	United States	Truven MarketScan Commercial Claims and Encounters Database	22,623		Overall ≈49% male (52.1% male in non-depressed vs. 32.4% in depressed cohort)		Elixhauser method: obesity, chronic kidney disease, alcohol use, tobacco use, hypertension, coronary artery disease, anxiety, congestive heart failure, rheumatic disease and diabetes	Primary TSA for glenohumeral disease	Anatomic (TSA) and reverse (rTSA)
Wilson et al 2021^84^	Pre-operative opioid use is an independent risk factor for complication, revision, and increased health care utilization following primary total shoulder arthroplasty.	JSES		United States	Administrative claims database	29,454				Elixhauser comorbidity categories		
Dacombe et al 2020^16^	Predictors of length of stay following shoulder arthroplasty in a high-volume UK center.	Annals of the royal college of Surgeons England	Retrospective consecutive cohort study	UK	Single high-volume UK institutional database (electronic patient records/case records)	640	Overall mean age 72 ± 10 yr	29.50%		Charlson Comorbidity Index, ASA Distribution	Osteoarthritis (297 cases), cuff-tear arthropathy (160), rheumatoid arthritis (36), avascular necrosis (18), trauma (22), trauma sequelae (49), infection (3), revision (55)	Anatomic total shoulder replacement (262), reverse shoulder arthroplasty (287), hemiarthroplasty (36), revision arthroplasty (55)
Menendez et al 2019^62^	Delayed hospital discharge after total shoulder arthroplasty: why, and who is at risk?	JSES Open Access	Retrospective cohort study	United States	Prospectively collected institutional registry with manual chart review	415	68.8 ± 8.4 yr	39%	30.8 ± 6.3 kg/m^2^	ASA class distribution, depression, diabetes	Elective primary anatomic or reverse TSA for degenerative shoulder conditions	Anatomic (TSA) and reverse (rTSA)
Biron et al 2020^7^	A Novel Machine Learning Model Developed to Assist in Patient Selection for Outpatient Total Shoulder Arthroplasty.	J Am Acad Orthop Surg	Retrospective cross sectional analysis	United States	ACS-NSQIP database	4,500	67.8 yr in short-stay vs. 72.8 yr in long-stay	51.1% male (1,084/2,122); long-stay: 31.9% male (321/1,006)	BMI 31.2 kg/m^2^ in short-stay vs. 31.7 kg/m^2^ long stay	ASA class, diabetes, CHF, COPD, hypertension, dyspnea, obesity	Elective total-shoulder arthroplasty	Anatomic and reverse TSA
Anthony et al 2019^3^	Utilization and Real-world Effectiveness of Tranexamic Use in Shoulder Arthroplasty: A Population-based Study.	Journal of the American Academy of Orthopedic Surgeons	Retrospective cohort study	United States	National claims database	82,512				Charlson-Deyo Comorbidity Index		
Lee et al 2019^47^	Low Serum Albumin Levels are Associated with Increased 30-d Cardiopulmonary Complications, Reoperation, and Readmission Rates Following Total Shoulder Arthroplasty.	IOWA Orthopedic Journal	Retrospective cohort study	United States	ACS-NSQIP database	14,494	Normal cohort 68.86 ± 9.82 yr; matched control 70.26 ± 9.44 yr; hypoalbuminemia 71.24 ± 10.39 yr	Normal cohort: 45.00% male; matched control: 34.43% male; hypoalbuminemia: 26.19% male		COPD 14.43% vs. 7.84%; CHF 2.27% vs. 0.62%; hypertension 72.99% vs. 69.07%; functional dependence 9.69% vs. 2.89%	Primary total shoulder arthroplasty	Anatomic and reverse TSA
Kim et al 2018^41^	Predicting adverse events, length of stay, and discharge disposition following shoulder arthroplasty: a comparison of the Elixhauser Comorbidity Measure and Charlson Comorbidity Index.	Seminars in Arthroplasty: JSES	Retrospective cohort study	United States	National Inpatient Sample (NIS), HCUP	90,491	69.2 ± 9.6 yr	41.80%	31.1 ± 6.6	Charlson Comorbidity Index (CCI) and Elixhauser Comorbidity Measure (ECM)	Elective primary TSA	Anatomic TSA and reverse TSA
Izquierdo-Fernández et al 2018^37^	Reverse shoulder arthroplasty in obese patients: analysis of functionality in the medium-term.	Archives of Orthopedic and Trauma Surgery	Retrospective cohort study	Spain	Single-center institutional database (University Hospital Reina Sofía, Spain)	29	78.1 yr	20.70%	31.85		Cuff-tear arthropathy and fractures/dislocations	Reverse shoulder arthroplasty
Fu et al 2017^25^	The impact of insulin dependence on short-term post-operative complications in diabetic patients undergoing total shoulder arthroplasty.	JSES	Retrospective cohort study	United States	ACS-NSQIP database	5,918	≤65 yr: Non-DM 28.6%, NIDDM 22.4%, IDDM 26.8% 66-75 yr: Non-DM 38.5%, NIDDM 45.7%, IDDM 42.0% >75 yr: Non-DM 32.9%, NIDDM 31.9%, IDDM 31.2% *P* values: Non-DM vs. NIDDM <.001; Non-DM vs. IDDM 0.478; NIDDM vs. IDDM 0.305	Female proportion: Non-DM 56.6%, NIDDM 53.8%, IDDM 56.3% *P* values: Non-DM vs. NIDDM 0.178; Non-DM vs. IDDM 0.926; NIDDM vs. IDDM 0.482	Non-obese (≤29.9 kg/m2): Non-DM 54.7%, NIDDM 32.3%, IDDM 26.4% Obese I (30-34.9): Non-DM 25.3%, NIDDM 30.7%, IDDM 27.1% Obese II (35-39.9): Non-DM 11.9%, NIDDM 20.7%, IDDM 22.7% Obese III (≥40): Non-DM 8.1%, NIDDM 16.4%, IDDM 23.7% *P* values: Non-DM vs. NIDDM <.001; Non-DM vs. IDDM <.001; NIDDM vs. IDDM 0.022	Modified CCI: 0-2: Non-DM 16.8%, NIDDM 1.6%, IDDM 3.1% 3: Non-DM 32.8%, NIDDM 8.0%, IDDM 11.9% 4: Non-DM 33.5%, NIDDM 37.3%, IDDM 29.5% ≥5: Non-DM 16.7%, NIDDM 52.8%, IDDM 55.6% *P* values: Non-DM vs. NIDDM <.001; Non-DM vs. IDDM <.001; NIDDM vs. IDDM 0.034 Functional status: Independent: Non-DM 96.4%, NIDDM 94.6%, IDDM 93.6% Non-independent: Non-DM 3.6%, NIDDM 5.4%, IDDM 6.4% *P* values: Non-DM vs. NIDDM 0.025; Non-DM vs. IDDM 0.013; NIDDM vs. IDDM 0.500 Individual comorbidities: Hypertension: Non-DM 63.0%, NIDDM 87.3%, IDDM 84.8% (*P* < .001, <.001, 0.705) Cardiac history: 2.1%, 4.6%, 6.8% (*P* < .001, <.001, 0.167) COPD: 5.9%, 9.4%, 10.2% (*P* < .001, .003, 0.710) Current smoker (within 1 yr): 10.3%, 7.8%, 10.9% (*P* = .041, .764, 0.122)		
Issa et al 2016^36^	Total Shoulder Arthroplasty Demographics, Incidence, and Complications—A Nationwide Inpatient Sample Database Study.	Surgical Technology International	Retrospective cohort study	United States	National Inpatient Sample (NIS), HCUP	226,227	68.9 (95% CI 68.77-68.96)	Male: 43.6%		0: 135,918 (60.01%) 1: 63,332 (27.96%) 2: 19,171 (8.46%) ≥3: 8,074 (3.56%)		
Weinreb et al 2016^83^	Shoulder Arthroplasty: Disposition and Perioperative Outcomes in Patients With and Without Rheumatoid Arthritis.	The American Journal of Orthopedics	Retrospective cohort study	United States	National Inpatient Sample (NIS), HCUP	34,970	RA: 66.39 ± 11.39 yr; Median 68 (IQR 60-75) Non-RA: 69.07 ± 9.99 yr; Median 70 (IQR 63-76) *P* < .001	RA: 24.49% Non-RA: 45.59% *P* < .001			Rheumatoid arthritis Non-RA indications not broken down	Reverse and traditional
Mollon et al 2016^64^	The influence of a history of clinical depression on peri-operative outcomes in elective total shoulder arthroplasty: a 10-yr national analysis.	The Bone & Joint Journal	Retrospective cohort study	United States	National Inpatient Sample (NIS), HCUP	224,060	With depression: 67.3 ± 9.6 yr Without depression: 69.5 ± 10.0 yr (*P* < .001) [Student's t-test]	With depression: 8,037 (28.7%) Without depression: 92,117 (47.0%) *P* < .001 (Fisher's exact test)		Gender Male With depression: 8,037 (8.0%) Without depression: 92,117 (92.0%) *P* < .001 Female With depression: 19,925 (16.0%) Without depression: 103,979 (84.0%) Income quartile < $39,000: 6,140 (13.0%) vs. 41,201 (87.0%) $39,000-$47,999: 7,631 (13.3%) vs. 49,922 (86.7%) $48,000-$62,999: 7,517 (12.5%) vs. 52,833 (87.5%) ≥ $63,000: 6,676 (11.4%) vs. 52,140 (88.6%) *P* < .001 Primary payer Medicare: 18,541 (12.0%) vs. 135,668 (88.0%) Medicaid: 758 (18.9%) vs. 3,252 (81.1%) Private: 7,493 (13.1%) vs. 49,872 (86.9%) Other: 1,172 (13.8%) vs. 7,304 (86.2%) *P* < .001 Ethnicity Caucasian: 25,475 (12.6%) vs. 176,209 (87.4%) African American: 940 (10.1%) vs. 8,340 (89.9%) Hispanic American: 863 (12.2%) vs. 6,192 (87.8%) Other: 686 (11.4%) vs. 5,355 (88.6%) *P* < .001		
Kandil et al 2016^39^	Blood transfusion after total shoulder arthroplasty: Which patients are at high risk?	International Journal of Shoulder Surgery	Retrospective cohort study	United States	National Inpatient Sample (NIS), HCUP	51,191	Any transfusion: 72.92 ± 10.33 yr No transfusion: 68.30 ± 11.31 yr Overall: 68.58 ± 11.31 yr	Any transfusion: 26.2% No transfusion: 45.6% Overall: 44.4%		Charlson–Deyo score: Transfusion: 0.57 ± 1.08 No transfusion: 0.28 ± 0.67 Overall: 0.03 ± 0.70 Individual comorbidities: Obesity: 8.9% vs. 9.8% (overall 9.7%) Smoking: 4.4% vs. 6.4% (overall 6.2%) Diabetes: 18.9% vs. 14.5% (overall 15.7%) Ischemic heart disease: 21.2% vs. 14.2% (overall 14.6%) CHF: 7.7% vs. 2.5% (overall 2.9%) COPD: 18.3% vs. 15.2% (overall 15.4%) RA: 2.3% vs. 2.9% (overall 2.9%) Depression: 11.5% vs. 8.7% (overall 8.9%) Hypertension (uncomplicated): 60.1% vs. 58.9% (overall 59.0%) Hematologic risk factors: Alcohol abuse: 1.0% vs. 0.7% (overall 0.8%) Liver disease: 3.5% vs. 0.9% (overall 1.1%) Coagulation disorders: 3.5% vs. 0.9% (overall 1.1%) Blood loss anemia: 34.8% vs. 5.0% (overall 7.5%) Deficiency anemia: 29.1% vs. 6.6% (overall 8.0%) All *P* < .001 other than Htn and alcohol abuse		
Murphy et al 2016^66^	Metabolic syndrome and shoulder arthroplasty: epidemiology and peri-operative outcomes.	International Orthopedics	Retrospective cohort study	United States	National Inpatient Sample (NIS), HCUP	422,371	With metabolic syndrome: 68.7 ± 11.7 yr Without metabolic syndrome: 69.4 ± 8.8 yr	With metabolic syndrome: 37.4% Without metabolic syndrome: 40.0%		Metabolic vs. no metabolic syndrome Congestive heart failure: 7.1% vs. 3.6% Chronic lung disease: 20.1% vs. 16.2% Liver disease: 1.0% vs. 1.0% Renal failure: 8.0% vs. 2.8% Peripheral vascular disease: 4.5% vs. 4.0% Ischemic heart disease: 7.2% vs. 4.2%	Metabolic vs. no metabolic syndrome Osteoarthritis: 61.9% vs. 61.3% Proximal humerus fracture: 19.1% vs. 16.3% Avascular necrosis: 1.8% vs. 3.6% Rheumatoid arthritis: 0.5% vs. 1.5% Non-union of humerus fracture: 2.5% vs. 2.2% Rotator cuff arthropathy: 9.9% vs. 9.9% Other: 4.2% vs. 5.2%	Total: 61.4 vs. 59.1 — 112 Partial: 38.6 vs. 40.9 — 103
Sivasundaram et al 2016^79^	Pre-operative risk factors for discharge to a postacute care facility after shoulder arthroplasty.	JSES	Retrospective cohort study	United States	National Inpatient Sample (NIS), HCUP	103,798	Overall: 69.7 ± 9.8 yr Routine discharge: 67.6 ± 9.6 PAC facility discharge: 76.8 ± 8.2 (*P* < .001)	Overall: 43.4% Routine discharge: 50.7% PAC discharge: 19.6% (*P* < .001)		Number of comorbidities: ≤3: 31.3% overall; 35.1% routine; 19.0% PAC 4-6: 44.1% overall; 43.9% routine; 42.8% PAC 7-9: 18.9% overall; 16.7% routine; 26.4% PAC ≥9: 5.8% overall; 4.3% routine; 11.9% PAC (*P* < .001) Individual comorbidities (multivariable analysis, ORs for PAC discharge): Hypertension: OR 0.95 (95% CI 0.84-1.08, *P* = .656) CHF: OR 2.00 (95% CI 1.44-2.77, *P* < .001) Diabetes: OR 1.51 (95% CI 1.29-1.77, *P* < .001) Peripheral vascular disease: OR 1.05 (95% CI 0.72-1.52, *P* = .240) CKD: OR 1.14 (95% CI 0.92-1.41, *P* = .238)	Osteoarthritis: 60.3% Rotator cuff arthropathy: 25.1% Humeral fractures: 11.7% Revision arthroplasties: 1.76% (1827 patients)	TSA: 56.8% overall; 62.9% routine; 36.6% PAC RTSA: 43.2% overall; 37.1% routine; 63.4% PAC (*P* < .001)
Garcia et al 2016^26^	Malnutrition: a marker for increased complications, mortality, and length of stay after total shoulder arthroplasty.	JSES	Retrospective cohort study	United States	National surgical quality database (ACS-NSQIP)	4,655	Normal albumin: 69.7 ± 10.3 yr Hypoalbuminemic: 73.0 ± 10.7 yr (*P* = .001)	Normal albumin: 42.9% male Hypoalbuminemic: 24.2% male (*P* < .001)	Normal albumin: 30.7 ± 6.7 Hypoalbuminemic: 31.2 ± 8.4 (*P* = .390)	Modified CCI ≥5 Normal: 24.8% Hypoalbuminemic: 50.0% (*P* < .001) ASA ≥3 Normal: 52.2% Hypoalbuminemic: 74.2% (*P* < .001) Individual comorbidities Hypertension Normal: 68.3% Hypoalbuminemic: 75.8% (*P* = .077) Cardiac comorbidityNormal: 4.8% Hypoalbuminemic: 10.2% (*P* = .008) Pulmonary comorbidity Normal: 12.6% Hypoalbuminemic: 28.9% (*P* < .001) Diabetes Normal: 17.3% Hypoalbuminemic: 29.7% (*P* < .001) Smoking Normal: 9.7% Hypoalbuminemic: 11.7% (*P* = .452) Peripheral vascular disease Normal: 0.2% Hypoalbuminemic: 0.8% (*P* = .190) TIA/CVA Normal: 2.7% Hypoalbuminemic: 6.3% (*P* = .023) Chronic steroid use Normal: 5.6% Hypoalbuminemic: 15.6% (*P* < .001)		
Basques et al 2015^5^	Length of Stay and Readmission After Total Shoulder Arthroplasty: An Analysis of 1505 Cases.	The American Journal of Orthopedics	Retrospective cohort study	United States	Institutional database (Rush University Medical Center)	1,505	60-69 yr: 558 (37.1%) 70-79 yr:625 (41.5%) ≥80 yr: 322 (21.4%)	Male sex: 41.1%	<30: 802 (53.3%) 30-35 (class I obesity): 400 (26.6%) 35-40 (class II obesity): 182 (12.1%) ≥40 (class III obesity): 121 (8.0%)	ASA class 3-4: 802 (53.3%) Individual comorbidities: Diabetes: 266 (17.7%) Smoking: 93 (6.2%) Pulmonary disease: 176 (11.7%) Heart disease: 122 (8.1%) Hypertension: 1,092 (72.6%) Steroid use: 80 (5.3%) Dependent functional status: 48 (3.2%)	Nonfracture: 1,445 (96.0%) Fracture-associated: 60 (4.0%)	Primary TSA only + reverse TSA (but wasn't separated)
Matsen et al 2015^59^	Factors Affecting Length of Stay, Readmission, and Revision After Shoulder Arthroplasty: A Population-Based Study.	The Journal of Bone and Joint Surgery	Retrospective cohort study	United States	Washington State Comprehensive Hospital Abstract Reporting System (CHARS)	17,311	Not given in mean or median	TSA: 43% HA: 30%		Charlson Comorbidity Index: 0: TSA 8,519; HA 7,542 1: TSA 262; HA 212 >1: TSA 42; HA 140 Specific comorbidities (TSA vs. HA): Hypertension: 5 vs. 4 Infection: 0 vs. 1 CNS: 211 vs. 294 Malignancy: 114 vs. 196 Endocrine: 1,238 vs. 1,114 Rheumatologic: 416 vs. 224 Overweight: 249 vs. 115 Substance abuse: 47 vs. 79 Pulmonary: 123 vs. 91 Cardiac: 951 vs. 802 GI: 202 vs. 152 Liver/kidney: 128 vs. 123	Fracture, inflammatory, osteoarthritis, trauma, cuff tear, osteonecrosis, miscellaneous	Primary TSA and HA
Pappou et al 2014^70^	Outcomes and Costs of Reverse Shoulder Arthroplasty in the Morbidly Obese: A Case Control Study.	The Journal of Bone and Joint Surgery	Retrospective cohort study	United States	Single-institution joint registry cohort	84	Morbidly obese: 69 ± 7 yr Controls: 71 ± 6 yr *P* = .152	Morbidly obese: 19% (4/21) Controls: 21% (13/63)	Morbidly obese: 43.2 ± 3.1 Controls: 27.1 ± 7.6 (*P* < .0001)	Charlson-Deyo Index (CDI): Morbidly obese: 2 ± 1 Controls: 1 ± 1 (*P* = .025) Total comorbidities (excluding obesity): Morbidly obese: 6 ± 2 Controls: 4 ± 2 (*P* = .001) Specific comorbidities: Obstructive sleep apnea (Yes vs. No): 10 vs. 2 (OR 27.7; 95% CI 5.3-144.1; *P* = .0001) Recent corticosteroid use: 4 vs. 5 (OR 2.73; 95% CI 0.7-11.3; *P* = .17)	Cuff tear arthropathy (CTA): 17 vs. 51 Massive rotator cuff tear (MRCT): 2 vs. 6 Rheumatoid arthritis (RA): 2 vs. 6 *P* = 1.0 (Fisher test)	Primary reverse shoulder arthroplasty
Ponce et al 2014^74^	Diabetes as a risk factor for poorer early post-operative outcomes after shoulder arthroplasty.	JSES	Retrospective database study	United States	National Inpatient Sample (NIS), HCUP	66,485	Overall: 69 ± 13 Diabetes: 70 ± 9.4 Non-diabetes: 69 ± 13 Uncomplicated diabetes: 70 ± 9.4 Complicated diabetes: 71 ± 9.4 Type 1 diabetes: 64 ± 12 Type 2 diabetes: 70 ± 9.3 *P* < .001 across groups	Overall: 43% Diabetes: 41% Non-diabetes: 43% Uncomplicated diabetes: 42% Complicated diabetes: 40% Type 1: 55% Type 2: 41% *P* = .004 (overall comparison)		Hypertension Overall: 67% Diabetes: 85% Nondiabetes: 63% *P* value: <.001 Diabetes sub-groups: Uncomplicated: 84% Complicated: 89% *P* value: <.001 Type 1: 71% Type 2: 85% *P* value: <.001 Congestive heart failure Overall: 3.8% Diabetes: 7.1% Nondiabetes: 2.9% *P* value: <.001 Diabetes sub-groups: Uncomplicated: 6.4% Complicated: 14% *P* value: <.001 Type 1: 7.2% Type 2: 7.1% *P* value: .95 Peripheral vascular disorders Overall: 2.6% Diabetes: 3.7% Nondiabetes: 2.3% *P* value: <.001 Diabetes sub-groups: Uncomplicated: 3.0% Complicated: 11% *P* value: <.001 Type 1: 3.6% Type 2: 3.7% *P* value: .94 Chronic pulmonary disease Overall: 17% Diabetes: 20% Nondiabetes: 16% *P* value: <.001 Diabetes sub-groups: Uncomplicated: 19% Complicated: 24% *P* value: <.001 Type 1: 17% Type 2: 20% *P* value: .45 Obesity Overall: 14% Diabetes: 25% Nondiabetes: 11% *P* value: <.001 Diabetes sub-groups: Uncomplicated: 23% Complicated: 38% *P* value: <.001 Type 1: 25% Type 2: 25% *P* value: .98 Hypothyroidism Overall: 15% Diabetes: 17% Nondiabetes: 14% *P* value: <.001 Diabetes sub-groups: Uncomplicated: 16% Complicated: 20% *P* value: <.001 Type 1: 27% Type 2: 17% *P* value: .001 Liver disease Overall: 1.1% Diabetes: 1.5% Nondiabetes: 1.0% *P* value: <.001 Diabetes sub-groups: Uncomplicated: 1.6% Complicated: 0.7% *P* value: .021 Type 1: 3.6% Type 2: 1.5% *P* value: .043 Renal failure Overall: 5.5% Diabetes: 11% Nondiabetes: 4.2% *P* value: <.001 Diabetes sub-groups: Uncomplicated: 8.9% Complicated: 29% *P* value: <.001 Type 1: 10% Type 2: 11% *P* value: .83 Depression Overall: 14% Diabetes: 16% Nondiabetes: 13% *P* value: <.001 Diabetes sub-groups: Uncomplicated: 16% Complicated: 19% *P* value: .005 Type 1: 27% Type 2: 16% *P* value: <.001 Deficiency anemias Overall: 10% Diabetes: 12% Nondiabetes: 9.7% *P* value: <.001 Diabetes sub-groups: Uncomplicated: 11% Complicated: 20% *P*-value: <.001 Type 1: 6.5% Type 2: 12% *P*-value: .049 Solid tumor without metastasis Overall: 0.6% Diabetes: 0.8% Nondiabetes: 0.5% *P*-value: <.001 Diabetes sub-groups: Uncomplicated: 0.8% Complicated: 0.8% *P*-value: .92 Type 1: 0% Type 2: 0.8% *P*-value: .29 Metastatic cancer Overall: 0.2% Diabetes: 0.2% Nondiabetes: 0.2% *P*-value: .85 Diabetes sub-groups: Uncomplicated: 0.2% Complicated: 0.7% *P*-value: <.001 Type 1: 0% Type 2: 0.2% *P*-value: .57		TSA, RTSA
Griffin et al 2014^27^	Morbid obesity in total shoulder arthroplasty: risk, outcomes, and cost analysis.	JSES	Retrospective cohort study	United States	National Inpatient Sample (NIS), HCUP	31,924	Non-obese: 68.3 ± 10.9 yr Obese: 66.8 ± 9.2 yr Morbidly obese: 64.9 ± 9.1 yr Total: 68.7 ± 10.8 yr *P* < .001	Non-obese: 44.5% male Obese: 35.2% male Morbidly obese: 31.6% male Total: 43.7% male *P* < .001		Deyo score: Non-obese: 0.28 ± 0.69 Obese: 0.28 ± 0.69 Morbidly obese: 0.38 ± 0.75 Total: 0.28 ± 0.69 *P* = .004 Number of diagnoses: Non-obese: 5.31 ± 3.06 Obese: 7.78 ± 3.17 Morbidly obese: 8.26 ± 3.36 Total: 5.51 ± 3.15 *P* < .001 Number of procedures: Non-obese: 1.52 ± 0.97 Obese: 1.54 ± 0.98 Morbidly obese: 1.60 ± 1.03 Total: 1.52 ± 0.98 *P* = .086 Specific comorbidity: Obstructive sleep apnea (OSA) Non-obese: 4.0% Obese: 15.6% Morbidly obese: 27.8% Total: 5.1% *P* < .001		
Johnson et al 2014^38^	Does pre-operative American Society of Anesthesiologists score relate to complications after total shoulder arthroplasty?	Clinical Orthopedics and Related Research	Retrospective single-surgeon cohort study	United States	Single-institution retrospective cohort	452	TSA: 63.9 ± 10.4 yr RTSA: 67.3 ± 12.2 yr Revision: 63.9 ± 10.7 yr *P* = .008 across groups	TSA: 55.8% male RTSA: 36.7% male Revision: 36.0% male *P* < .001	TSA: 28.8 ± 4.9 RTSA: 29.8 ± 6.8 Revision: 31.6 ± 7.1 *P* = .008	Charlson Comorbidity Index: TSA: 1.3 ± 2.0 RTSA: 1.3 ± 1.7 Revision: 1.2 ± 1.5 *P* = .900 ASA score distribution: TSA: ASA I 5 (2.2%) ASA II 155 (69.2%) ASA III 64 (28.6%) ASA IV 0 RTSA: ASA I 4 (2.4%) ASA II 80 (47.6%) ASA III 82 (48.8%) ASA IV 2 (1.2%) Revision: ASA I 0 ASA II 33 (64.7%) ASA III 18 (35.3%) ASA IV 0 *P* = .001 Allergies (median, IQR): TSA: 0 (0-1) RTSA: 0 (0-1) Revision: 0.5 (0-1) *P* = .736 Medications (median, IQR): TSA: 5 (3-8) RTSA: 7 (4-10) Revision: 5 (2-7) *P* < .001	Diagnosis – *P* < .001 Osteoarthritis TSA: 206 (91.6%) RTSA: 75 (42.6%) Failed arthroplasty TSA: 0 RTSA: 5 (2.8%) Cuff arthropathy TSA: 0 RTSA: 50 (28.2%) Infection TSA: 0 RTSA: 7 (4.0%) Instability TSA: 1 (0.4%) RTSA: 5 (2.8%) Rheumatoid arthritis TSA: 3 (1.3%) RTSA: 12 (6.8%) Osteonecrosis TSA: 11 (4.9%) RTSA: 3 (1.7%) Fracture TSA: 1 (0.4%) RTSA: 6 (3.4%) Cuff tear with pseudoparalysis TSA: 1 (0.4%) RTSA: 5 (2.8%) Traumatic arthritis/arthritis of dislocation TSA: 0 RTSA: 8 (4.5%)	Total shoulder arthroplasty and RTSA
Griffin et al 2013^28^	Obstructive sleep apnea as a risk factor after shoulder arthroplasty.	JSES	Retrospective cohort study	United States	National Inpatient Sample (NIS), HCUP	22,988	Overall: 68.81 ± 11.59 yr No OSA: 68.99 ± 11.7 yr OSA: 66.06 ± 9.48 yr (*P* = .000)	39.70%		Deyo score: Overall: 0.35 ± 0.84 No OSA: 0.35 ± 0.04 OSA: 0.44 ± 0.03 (*P* = .000) Distribution: 0 = 76.2% 1 = 17.0% 2 = 4.7% ≥3 = 1.0%		TSA and hemiarthroplasty
Humphries et al 2008^35^	Effect of the Deyo score on outcomes and costs in shoulder arthroplasty patients.	Journal of Orthopedic Surgery	Retrospective database cohort	United States	National Inpatient Sample (NIS), HCUP	28,434	No mean given (only age groups)	33.50%		Deyo index (0, 1-4, 5-36)		
Elsabbagh et al 2025^21^	Does use of glucagon-like peptide-1 agonists increase perioperative complications in patients undergoing shoulder arthroplasty?	JSES	Retrospective Cohort Study	United States	National database (exact database not specified in provided information)	64,567	90-d matched: GLP-1 = 68.2 ± 6.8; Non-GLP-1 = 68.3 ± 6.7 *P* = .269 2-yr matched: GLP-1 = 67.8 ± 6.5; Non-GLP-1 = 67.9 ± 6.5 *P* = .302	90-d matched: GLP-1 = 45.1%; Non-GLP-1 = 45.0% 2-yr matched: GLP-1 = 45.0%; Non-GLP-1 = 44.7%		90-d matched GLP-1 = 2.7 ± 1.9; Non-GLP-1 = 2.6 ± 1.8 (*P* = .006) Congestive heart failure: 1845 (9.9%) vs. 460 (9.2%); *P* = .154 Arrhythmias: 4154 (22.2%) vs. 1090 (21.8%); *P* = .502 Valvular disease: 3620 (19.4%) vs. 966 (19.3%); *P* = .920 Pulmonary circulatory disorders: 1224 (6.5%) vs. 357 (7.1%); *P* = .152 Peripheral vascular disease: 4005 (21.4%) vs. 1015 (20.3%); *P* = .078 Hypertension: 16,521 (88.3%) vs. 4511 (90.0%); *P* < .001 Paralysis: 302 (1.6%) vs. 77 (1.5%); *P* = .743 Other neuro disorders: 1064 (5.7%) vs. 235 (4.7%); *P* = .006 Chronic pulmonary disease: 6300 (33.7%) vs. 1671 (33.4%); *P* = .668 Hypothyroidism: 5162 (27.6%) vs. 1371 (27.4%); *P* = .752 Chronic kidney disease: 3120 (16.7%) vs. 916 (18.3%); *P* = .008 Liver disease: 2632 (14.1%) vs. 742 (14.8%); *P* = .193 Peptic ulcer disease: 909 (4.9%) vs. 229 (4.6%); *P* = .415 Lymphoma: 198 (1.1%) vs. 54 (1.1%); *P* = .969 Metastatic cancer: 185 (1.0%) vs. 51 (1.0%); *P* = .919 Nonmetastatic cancer: 2265 (12.1%) vs. 619 (12.4%); *P* = .657 Rheumatoid arthritis: 1218 (6.5%) vs. 293 (5.8%); *P* = .093 Coagulopathy: 1268 (6.8%) vs. 308 (6.1%); *P* = .118 Fluid and electrolyte disorders: 4951 (26.5%) vs. 1227 (24.5%); *P* = .005 Blood loss anemia: 677 (3.6%) vs. 163 (3.3%); *P* = .229 Deficiency anemia: 2993 (16.0%) vs. 742 (14.8%); *P* = .041 Alcohol abuse: 634 (3.4%) vs. 110 (2.2%); *P* < .001 Drug abuse: 1202 (6.4%) vs. 236 (4.7%); *P* < .001 Psychoses: 540 (2.9%) vs. 107 (2.1%); *P* = .004 Depression: 6255 (33.4%) vs. 1738 (34.7%); *P* = .102 Smoking: 4613 (24.7%) vs. 1289 (25.7%); *P* = .127 Obesity: 8165 (43.7%) vs. 2613 (52.2%); *P* < .001 Metformin use: 12,880 (68.9%) vs. 3471 (69.3%); *P* = .591 Insulin use: 2859 (15.3%) vs. 899 (17.9%); *P* < .001 Complicated diabetes: 8983 (48.0%) vs. 2487 (49.6%); *P* = .045 Osteoporosis: 2421 (12.9%) vs. 561 (11.2%); *P* = .001 Chronic steroid use: 552 (3.0%) vs. 119 (2.4%); *P* = .033 Steroid-induced diabetes: 48 (0.3%) vs. 10 (0.2%); *P* = .572 2-y matched GLP-1 = 2.6 ± 1.8; Non-GLP-1 = 2.5 ± 1.7 (*P* = .004) Congestive heart failure: 1122 (8.8%) vs. 292 (8.5%); *P* = .528 Arrhythmias: 2422 (19.1%) vs. 642 (18.6%); *P* = .574 Valvular disease: 2119 (16.7%) vs. 570 (16.6%); *P* = .860 Pulmonary circulatory disorders: 676 (5.3%) vs. 198 (5.7%); *P* = .352 Peripheral vascular disease: 2358 (18.6%) vs. 633 (18.4%); *P* = .809 Hypertension: 10,959 (86.3%) vs. 3031 (88.0%); *P* = .012 Paralysis: 184 (1.4%) vs. 46 (1.3%); *P* = .675 Other neuro disorders: 614 (4.8%) vs. 135 (3.9%); *P* = .026 Chronic pulmonary disease: 3967 (31.3%) vs. 1066 (31.0%); *P* = .749 Hypothyroidism: 3237 (25.5%) vs. 901 (26.2%); *P* = .447 Chronic kidney disease: 1705 (13.4%) vs. 528 (15.3%); *P* = .005 Liver disease: 1465 (11.5%) vs. 440 (12.8%); *P* = .050 Peptic ulcer disease: 523 (4.1%) vs. 124 (3.6%); *P* = .183 Lymphoma: 114 (0.9%) vs. 28 (0.8%); *P* = .710 Metastatic cancer: 99 (0.8%) vs. 24 (0.7%); *P* = .699 Nonmetastatic cancer: 1402 (11.0%) vs. 374 (10.9%); *P* = .779 Rheumatoid arthritis: 812 (6.4%) vs. 162 (4.7%); *P* < .001 Coagulopathy: 705 (5.6%) vs. 176 (5.1%); *P* = .329 Fluid and electrolyte disorders: 2844 (22.4%) vs. 738 (21.4%); *P* = .229 Blood loss anemia: 382 (3.0%) vs. 102 (3.0%); *P* = .928 Deficiency anemia: 1776 (14.0%) vs. 411 (11.9%); *P* = .002 Alcohol abuse: 314 (2.5%) vs. 71 (2.1%); *P* = .179 Drug abuse: 622 (4.9%) vs. 125 (3.6%); *P* = .002 Psychoses: 318 (2.5%) vs. 59 (1.7%); *P* = .008 Depression: 3796 (29.9%) vs. 1057 (30.7%); *P* = .386 Smoking: 2428 (19.1%) vs. 704 (20.4%); *P* = .089 Obesity: 4822 (38.0%) vs. 1593 (46.3%); *P* < .001 Metformin use: 8122 (64.0%) vs. 2224 (64.6%); *P* = .540 Insulin use: 1512 (11.9%) vs. 508 (14.8%); *P* < .001 Complicated diabetes: 4924 (38.8%) vs. 1409 (40.9%); *P* = .025 Osteoporosis: 1534 (12.1%) vs. 363 (10.5%); *P* = .014 Chronic steroid use: 292 (2.3%) vs. 57 (1.7%); *P* = .025 Steroid-induced diabetes: 20 (0.2%) vs. 5 (0.1%); *P* = 1.000	Osteoarthritis	Primary TSA (no differentiation between anatomic and reverse TSA)
Alder et al 2024^2^	Increasing severity of pre-operative anemia is associated with higher post-operative medical and surgical complications after primary shoulder arthroplasty.	JSES	Retrospective Cohort Study	United States	Single-institution shoulder arthroplasty registry	5,231	No anemia: 68.8 ± 10.8 yr Mild anemia: 72.0 ± 10.4 yr Moderate-to-severe anemia: 70.9 ± 13.0 yr P(no anemia vs. mild anemia) <0.001 P(no anemia vs. moderate-to-severe anemia) = 0.003 P(mild anemia vs. moderate-to-severe anemia) = 0.318	No anemia: 48.2% male Mild anemia: 48.9% male Moderate-to-severe anemia: 28.8% male P(no anemia vs. mild anemia) = 0.712 P(no anemia vs. moderate-to-severe anemia) < 0.001 P(mild anemia vs. moderate-to-severe anemia) < 0.001 These *P* values are for the female sex	No anemia: 31.0 ± 9.3 Mild anemia: 30.2 ± 6.8 Moderate-to-severe anemia: 29.9 ± 7.2 P(no anemia vs. mild anemia) = 0.092 P(mild anemia vs. moderate-to-severe anemia) = 0.117 P(mild anemia vs. moderate-to-severe anemia) = 0.858	P(no anemia vs. mild anemia)/P(mild anemia vs. moderate-to-severe anemia)/P(mild anemia vs. moderate-to-severe anemia) Tobacco use No anemia: 339 (8.1%) Mild anemia: 46 (6.2%) Moderate-to-severe anemia: 20 (6.8%) *P* values: .290/.303/.328 Myocardial infarction (MI) No anemia: 49 (1.2%) Mild anemia: 17 (2.3%) Moderate-to-severe anemia: 9 (3.1%) *P* values: .014/.006/.480 Congestive heart failure (CHF) No anemia: 134 (3.2%) Mild anemia: 56 (7.5%) Moderate-to-severe anemia: 28 (9.5%) *P* values: <.001/<.001/.301 Peripheral vascular disease (PVD) No anemia: 216 (5.2%) Mild anemia: 54 (7.3%) Moderate-to-severe anemia: 27 (9.2%) *P* values: .019/.003/.310 Cerebrovascular accident (CVA) No anemia: 99 (2.4%) Mild anemia: 28 (3.8%) Moderate-to-severe anemia: 11 (3.7%) *P* values: .025/.142/.973 Dementia No anemia: 30 (0.7%) Mild anemia: 5 (0.7%) Moderate-to-severe anemia: 0 (0.0%) *P* values: .901/.145/.158 Chronic obstructive pulmonary disease (COPD) No anemia: 310 (7.4%) Mild anemia: 69 (9.3%) Moderate-to-severe anemia: 43 (14.6%) *P* values: .072/<.001/.014 Diabetes mellitus No anemia: 389 (9.3%) Mild anemia: 107 (14.4%) Moderate-to-severe anemia: 47 (15.9%) *P* values: <.001/<.001/.537 Chronic kidney disease (CKD) No anemia: 148 (3.5%) Mild anemia: 57 (7.7%) Moderate-to-severe anemia: 43 (14.6%) *P* values: <.001/<.001/<.001 ASA score (mean ± SD) No anemia: 2.3 ± 0.6 Mild anemia: 2.5 ± 0.6 Moderate-to-severe anemia: 2.6 ± 0.7 *P* values: <.001/<.001/.852 ASA class ≤2/≥3 ≤2: 2581/341/124 ≥3: 1613/401/171 *P* values: <.001/<.001/.252 Charlson Comorbidity Index (CCI), mean ± SD No anemia: 3.1 ± 1.8 Mild anemia: 3.8 ± 2.2 Moderate-to-severe anemia: 4.1 ± 2.6 *P* values: <.001/<.001/.052 CCI >2 No anemia: 2613 (62.3%) Mild anemia: 554 (74.7%) Moderate-to-severe anemia: 226 (76.6%) *P* values: <.001/<.001/.512 CCI class 0/1-3/4-5/≥6 0: 164/16/12 1-3: 2680/360/129 4-5: 1021/244/87 ≥6: 329/122/67 *P* values: <.001/<.001/.027 Pre-operative hemoglobin (g/dL) No anemia: 14.0 ± 1.2 Mild anemia: 12.0 ± 0.8 Moderate-to-severe anemia: 9.9 ± 1.0 *P* values: <.001/<.001/<.001 Pre-operative hematocrit (%) No anemia: 41.8 ± 3.1 Mild anemia: 35.8 ± 1.7 Moderate-to-severe anemia: 30.1 ± 2.7 *P* values: <.001/<.001/<.001	All *P* values <.001 Osteoarthritis: No anemia 50.9%; Mild anemia 42.0%; Moderate-to-severe anemia 32.9% Rotator cuff tear arthropathy: No anemia 31.6%; Mild anemia 35.3%; Moderate-to-severe anemia 26.4% PTOA: No anemia 9.5%; Mild anemia 9.0%; Moderate-to-severe anemia 6.8% AVN: No anemia 2.8%; Mild anemia 3.2%; Moderate-to-severe anemia 4.1% Inflammatory arthritis: No anemia 2.3%; Mild anemia 3.6%; Moderate-to-severe anemia 5.4% Acute fracture: No anemia 1.8%; Mild anemia 5.0%; Moderate-to-severe anemia 22.7% Other: No anemia 1.0%; Mild anemia 1.8%; Moderate-to-severe anemia 1.7%	All *P* values <.001 Hemiarthroplasty: No anemia 9.5%; Mild anemia 15.1%; Moderate-to-severe anemia 21.4% aTSA: No anemia 46.2%; Mild anemia 48.2%; Moderate-to-severe anemia 53.9% rTSA: No anemia 44.3%; Mild anemia 36.7%; Moderate-to-severe anemia 24.7%
Khokhar et al 2024^40^	Does morbid obesity negatively impact perioperative outcomes following elective reverse shoulder arthroplasty?: a propensity-matched comparative study.	Journal of Shoulder and Elbow Surgery International	Retrospective Cohort Comparison	United States	Single-institution retrospective propensity-matched cohort	175	Overall: 71 (66, 76) Morbidly obese: 68 (64, 73) Non-morbidly obese (full cohort): 71 (66.5, 77) Non-matched *P* = 0.12 Matched non-morbidly obese: 68 (65, 73) Matched p: 0.80	Morbidly obese: 16% Non-morbidly obese: 26% Matched non-morbidly obese: 10%	Morbidly obese: 42.4 (41.0, 45.1) Non-morbidly obese: 30.3 (27.2, 33.1) Matched non-morbidly obese: 30.6 (26.4, 32.7)	Overall cohort (n = 175) mFI-5 Score 0-1 BMI ≥40: 8 (42%) BMI <40: 76 (49%) *P* = .59 Score ≥2 BMI ≥40: 11 (58%) BMI <40: 80 (51%) Smoking status Never BMI ≥40: 6 (32%) BMI <40: 87 (56%) *P* = .10 Former BMI ≥40: 11 (58%) BMI <40: 53 (34%) Current BMI ≥40: 2 (10%) BMI <40: 16 (10%) Prior ipsilateral surgery None BMI ≥40: 16 (84%) BMI <40: 112 (72%) *P* = .85 Arthroscopy BMI ≥40: 3 (16%) BMI <40: 35 (22%) Open surgery BMI ≥40: 0 (0%) BMI <40: 5 (3%) Open surgery/arthroscopy BMI ≥40: 0 (0%) BMI <40: 4 (3%) Age-adjusted Charlson comorbidity index (ACCI) BMI ≥40: 4 (IQR 3-5) BMI <40: 4 (IQR 3-5) *P* = .88 Propensity matched cohort (n = 60) mFI-5 Score 0-1 BMI ≥40: 8 (42%) BMI <40: 14 (34%) *P* = .55 Score ≥2 BMI ≥40: 11 (58%) BMI <40: 27 (56%) Smoking status Never BMI ≥40: 6 (32%) BMI <40: 23 (56%) *P* = .19 Former BMI ≥40: 11 (58%) BMI <40: 14 (34%) Current BMI ≥40: 2 (10%) BMI <40: 4 (10%) Prior ipsilateral surgery None BMI ≥40: 16 (84%) BMI <40: 24 (59%) *P* = .25 Arthroscopy BMI ≥40: 3 (16%) BMI <40: 12 (29%) Open surgery BMI ≥40: 0 (0%) BMI <40: 3 (7%) Open surgery/arthroscopy BMI ≥40: 0 (0%) BMI <40: 2 (5%) Age-adjusted Charlson comorbidity index (ACCI) BMI ≥40: 4 (IQR 3-5) BMI <40: 4 (IQR 3-6) *P* = .75	RTC-deficient arthritis, GH OA, Chronic RTC tear, Other (this is what the table says)	Primary rTSA
Destine et al 2024^17^	Effect of hospital length of stay on functional outcomes and readmission after total shoulder arthroplasty.	JSES	Retrospective Cohort Study	United States	Single-institution retrospective cohort (Thomas Jefferson University Hospital)	217	Overall: 67.5 ± 8.85 yr >24 h: 70.1 ± 10.4 yr <24 h: 66.7 ± 8.10 yr (*P* = .030)	Overall: 98 (45.2%) >24 h: 24 (42.9%) <24 h: 74 (46.0%) (*P* = .805)	Overall: 30.4 ± 5.00 >24 h: 31.1 ± 3.83 <24 h: 30.2 ± 5.33 (*P* = .108)	Charlson comorbidity index (CCI) - group characteristics by LOS Overall: 3.10 ± 1.64 >24 h: 4.02 ± 2.07 <24 h: 2.78 ± 1.33 (*P* = .001) Group characteristic by CCI thresholds CCI <3 - 1.62 ± 0.58 CCI ≥3 - 4.08 ± 1.36 *P* < .001 CCI <4 - 2.19 ± 0.81 CCI ≥4 - 5.04 ± 1.23 *P* < .001		rTSA and aTSA
Moore et al 2024^65^	Fibromyalgia syndrome is an independent predictor of increased complications, revision, and cost of admission within 180 d of total shoulder arthroplasty.	JSES	Retrospective matched cohort study	United States	National readmissions database	11,012	FMS: 68 ± 8.2 yr No FMS: 67 ± 8.0 yr (*P* = .506)	6% in both groups		Charlson-Deyo Comorbidity Index (CCI): FMS 1.4 ± 1.6 vs. No FMS 1.4 ± 1.6 (*P* = .875) Hypertension requiring medication FMS: 72% No FMS: 70% *P* = .022 † Depression FMS: 37% No FMS: 22% *P* < .001 ∗ Diabetes mellitus (DM) FMS: 15% No FMS: 22% *P* < .001 ∗ Congestive heart failure (CHF) FMS: 5.7% No FMS: 4.5% *P* = .005 † Peripheral vascular disease (PVD) FMS: 3.7% No FMS: 3.1% *P* = .102 Chronic kidney disease (CKD) FMS: 12% No FMS: 9.8% *P* = .002 ∗ Chronic lung disease FMS: 34% No FMS: 33% *P* = .048 † Chronic liver disease FMS: 2.9% No FMS: 3.6% *P* = .024 † Key: ∗ Statistically significant † Not significant following Bonferroni correction		Primary anatomy and reverse TSA
Miltenberg et al 2024^63^	Prediction of overnight stay following shoulder arthroplasty utilizing machine learning.	Journal of Shoulder and Elbow Surgery	Retrospective cohort study	United States	ACS-NSQIP database	5,811	18-60 yr: OHS 9.72% vs. SHS 15.66% 61-75 yr: OHS 51.30% vs. SHS 59.80% 76+ yr: OHS 39.98% vs. SHS 24.54% *P* < .0001 Multivariable *P* = .004	OHS: 30.67% SHS: 47.82% *P* < .0001 Same value for multivariable P	<25: OHS 21.17%, SHS 16.85% 25-30: OHS 32.61%, SHS 38.89% 31-35: OHS 21.38%, SHS 24.34% 36+: OHS 24.84%, SHS 19.92% *P* < .0001 Multivariable *P* = .012	OHS vs. SHS Anemia: 32.94% vs. 24.46%, *P* < .0001, multivariable *P* < .0001, OR 1.43 (2.21-1.69), +43% risk ASA grade 3-5: 72.25% vs. 54.86%, *P* < .0001, multivariable *P* < .0001, OR 1.88 (1.58-2.23), +88% risk CHF: 5.40% vs. 2.99%, *P* = .0002 COPD: 10.69% vs. 5.08%, *P* < .0001, multivariable *P* < .0001, OR 1.89 (1.44-2.47) Dependent functional status: 6.59% vs. 1.54%, *P* < .0001, multivariable *P* < .0001, OR 3.28 (2.24-4.81), +228% risk Diabetes mellitus: 23.11% vs. 17.48%, *P* < .0001, multivariable *P* < .0001, OR 2.85 (1.88-3.72), +185% risk Hypertension: 68.47% vs. 63.95%, *P* = .008 Smoking history: 9.29% vs. 9.09%, *P* = .848		Primary elective TSA
Kopriva et al 2024^45^	Tramadol use before total shoulder arthroplasty: patients have lower risk of complications and resource utilization than those using traditional opioids.	Journal of Shoulder and Elbow Surgery International	Retrospective cohort study	United States	National claims database	29,454	Age distribution by opioid cohort (*P* < .001) <55 yr Opioid naïve: 1317 (8.8%) Tramadol: 155 (7.0%) Traditional opioids: 1082 (10.3%) Opioids + tramadol: 202 (11.1%) 55-64 yr Opioid naïve: 5230 (35.0%) Tramadol: 770 (34.8%) Traditional opioids: 3983 (38.1%) Opioids + tramadol: 729 (39.9%) 65-74 yr Opioid naïve: 4431 (29.6%) Tramadol: 656 (29.6%) Traditional opioids: 2942 (28.1%) Opioids + tramadol: 477 (26.1%) 75-84 yr Opioid naïve: 4431 (23.3%) Tramadol: 548 (24.8%) Traditional opioids: 2151 (20.6%) Opioids + tramadol: 361 (19.8%) 85+ yr Opioid naïve: 495 (3.3%) Tramadol: 85 (3.8%) Traditional opioids: 304 (2.9%) Opioids + tramadol: 56 (3.1%)	Opioid naïve: 53.4% Tramadol: 39.2% Traditional opioids: 47.0% Opioids and tramadol: 40.6%		**Obesity (*P* = .002)** Opioid naïve: 1651 (11.0%) Tramadol: 275 (12.4%) Traditional opioids: 1283 (12.3%) Opioids + tramadol: 241 (13.2%) **Chronic kidney disease (*P* < .001)** Opioid naïve: 672 (4.5%) Tramadol: 138 (6.2%) Traditional opioids: 646 (6.2%) Opioids + tramadol: 109 (6.0%) **Alcohol use disorders (*P* < .001)** Opioid naïve: 105 (0.7%) Tramadol: 23 (1.0%) Traditional opioids: 124 (1.2%) Opioids + tramadol: 20 (1.1%) Tobacco use (*P* < .001) Opioid naïve: 453 (3.0%) Tramadol: 75 (3.4%) Traditional opioids: 500 (4.8%) Opioids + tramadol: 90 (4.9%) **Hypertension (*P* < .001)** Opioid naïve: 8719 (58.3%) Tramadol: 1411 (63.4%) Traditional opioids: 6526 (62.3%) Opioids + tramadol: 1180 (64.7%) **Coronary artery disease (*P* < .001)** Opioid naïve: 2411 (16.1%) Tramadol: 397 (17.9%) Traditional opioids: 1846 (17.6%) Opioids + tramadol: 328 (18.0%) **Congestive heart failure (*P* < .001)** Opioid naïve: 557 (3.7%) Tramadol: 95 (4.3%) Traditional opioids: 585 (5.6%) Opioids + tramadol: 84 (4.6%) **Rheumatic disease (*P* < .001)** Opioid naïve: 612 (4.1%) Tramadol: 158 (7.1%) Traditional opioids: 696 (6.7%) Opioids + tramadol: 130 (7.1%) **Diabetes (*P* < .001)** Opioid naïve: 2870 (19.2%) Tramadol: 446 (20.1%) Traditional opioids: 2355 (22.5%) Opioids + tramadol: 391 (21.4%) **Depression (*P* < .001)** Opioid naïve: 1188 (7.9%) Tramadol: 203 (9.2%) Traditional opioids: 1461 (13.9%) Opioids + tramadol: 302 (16.6%)		
Kim et al 2024^43^	Cumulative effect of chronic dehydration and age on post-operative complications after total shoulder arthroplasty.	Journal of Shoulder and Elbow Surgery International	Retrospective cohort study	United States	ACS-NSQIP database (2015-2019)	16,993		Non-dehydrated: 52.53% Moderately dehydrated: 38.92% Severely dehydrated: 23.17% *P* < .001	<18.5: 0.69%-0.95% 18.5-29.9: ∼47% 30-34.9: ∼25%-27% 35-39.9: ∼14% ≥40: 10%-12%	Main Cohort ASA classification (*P* = .028) 1-2: 3636 (42.79%) ≥3: 4862 (57.21%) Functional status (*P* < .001) Independent: 8355 (98.32%) Dependent: 143 (1.68%) Smoking (*P* < .001) No: 7311 (86.03%) Yes: 1187 (13.97%) Outpatient/inpatient (*P* = .632) Outpatient: 791 (9.31%) Inpatient: 7707 (90.69%) Diabetes No: 7078 (83.29%) (*P* = .012) Non-insulin: 1047 (12.32%) (*P* = .028) Insulin: 373 (4.39%) (*P* = .021) Dyspnea No: 7942 (93.46%) (*P* = .206) Moderate exertion: 529 (6.22%) (*P* = .354) At rest: 27 (0.32%) (*P* = .652) COPD (*P* = .004) No: 7858 (92.47%) Yes: 640 (7.53%) Ascites (*P* = .154) No: 8498 (100.00%) Yes: 0 (0.00%) Congestive heart failure (*P* = .010) No: 8464 (99.60%) Yes: 34 (0.40%) Hypertension (*P* < .001) No: 2926 (34.43%) Yes: 5572 (65.57%) Disseminated cancer (*P* = .649) No: 8480 (99.79%) Yes: 18 (0.21%) Chronic steroid use (*P* = .007) No: 8128 (95.65%) Yes: 370 (4.35%) Bleeding disorders (*P* = .632) No: 8286 (97.51%) Yes: 212 (2.49%) Elderly Cohort Functional status (*P* = .034) Independent: 4594 (97.97%) Dependent: 95 (2.03%) Smoking (*P* < .001) No: 4352 (92.81%) Yes: 337 (7.19%) Outpatient/inpatient (*P* = .357) Outpatient: 366 (7.81%) Inpatient: 4323 (92.19%) Diabetes No: 3851 (82.13%) (*P* = .009) Non-insulin: 622 (13.27%) (*P* = .137) Insulin: 216 (4.61%) (*P* = .012) Dyspnea No: 4351 (92.79%) (*P* = .119) Moderate exertion: 321 (6.85%) (*P* = .193) At rest: 17 (0.36%) (*P* = .913) COPD (*P* = .030) No: 4321 (92.15%) Yes: 368 (7.85%) Ascites (p = –) No: 4689 (100.00%) Yes: 0 (0.00%) Congestive heart failure (*P* = .012) No: 4663 (99.45%) Yes: 26 (0.55%) Hypertension (*P* < .001) No: 1326 (28.28%) Yes: 3363 (71.72%) Disseminated cancer (*P* = .578) No: 4679 (99.79%) Yes: 10 (0.21%) Chronic steroid use (*P* = .437) No: 4480 (95.54%) Yes: 209 (4.46%) Bleeding disorders (*P* = .312) No: 4555 (97.14%) Yes: 134 (2.86%)		
Hartwell et al 2019^31^	Total shoulder arthroplasty: Risk factors for a prolonged length of stay. A retrospective cohort study.	Current Orthopedic Practice	Retrospective database study	United States	ACS-NSQIP database	14,339	Age (mean ± SD): Overall: 69.0 ± 9.7 yr LOS ≤24 h: 67.4 ± 9.7 LOS >24 h: 70.3 ± 9.7 *P* < .0001	% male: Overall: 44.0% LOS ≤24 h: 54.7% LOS >24 h: 45.3%	Overall: 31.1 ± 6.9 LOS ≤24 h: 31.0 ± 6.5 LOS >24 h: 31.1 ± 7.1	Body Mass Index (BMI): LOS ≤24 hr: 31.0 ± 6.5 LOS >24 hr: 31.1 ± 7.1, *P* = .29 Diabetes: LOS ≤24 hr: 233 (29.1%) LOS >24 hr: 568 (70.9%), *P* < .0001 Smoking: LOS ≤24 hr: 730 (48.3%) LOS >24 hr: 781 (51.7%), *P* = .016 Chronic obstructive pulmonary disease (COPD): LOS ≤24 hr: 299 (32.5%) LOS >24 hr: 620 (67.5%), *P* < .0001 Congestive heart failure (CHF): LOS ≤24 hr: 10 (14.7%) LOS >24 hr: 58 (85.3%), *P* < .0001 Hypertension: LOS ≤24 hr: 4097 (42.7%) LOS >24 hr: 5489 (57.3%), *P* < .0001 Dialysis: LOS ≤24 hr: 12 (19.7%) LOS >24 hr: 49 (80.3%), *P* < .0001 Steroids: LOS ≤24 hr: 284 (40.3%) LOS >24 hr: 421 (59.7%), *P* = .0059 Bleeding disorders: LOS ≤24 hr: 127 (33.0%) LOS >24 hr: 258 (67.0%), *P* < .0001 American Society of Anesthesiologists (ASA classification, mean ± SD): LOS ≤24 hr: 2.4 ± 0.6 LOS >24 hr: 2.6 ± 0.6, *P* < .0001		
Lonergan et al 2019^56^	Mood disorders do not predict a more difficult course after primary anatomic total shoulder arthroplasty.	Current Orthopedic Practice	Retrospective cohort study	United States	Prospectively collected institutional cohort	133	Mood disorder: mean 60.8 yr (range 30-70). No mood disorder: mean 62.9 yr (range 27-83)	Mood disorder: 8/29 (28%). No mood disorder: 60/97 (62%)	Mood disorder: 35.1 (no SD reported) No mood disorder: 33.0 (no SD reported)	Pre-op narcotic use	Primary osteoarthritis, inflammatory arthritis, osteonecrosis, capsulorrhaphy, arthropathy, posttraumatic arthritis of shoulder	Primary anatomic TSA
Hammond et al 2003^30^	Surgeon Experience and Clinical and Economic Outcomes for Shoulder Arthroplasty.	The Journal of Bone and Joint Surgery	Retrospective cohort study	United States	New York State SPARCS database	1,868	Mean - 68.1	31.8		CCI	Pertinent diagnoses assessed were OA, RA, and **fractures of the humerus**	
Lee et al 2020^47^	Pre-operative hyponatremia is associated with post-operative major morbidity, prolonged length of stay, non-home discharge, and readmission in total shoulder arthroplasty.	Journal of Shoulder and Elbow Surgery	Retrospective cohort study	United States	ACS-NSQIP database	12,437	Age: Overall: 69.4 ± 9.6 yr Hyponatraemia: 71.4 ± 9.6 yr Normonatraemia: 69.3 ± 9.6 yr *P* < .001	% male: Overall: 43.6%. Hyponatraemia: 33.3%. Normonatraemia: 44.4%. *P* < .001	BMI (mean ± SD): Overall: 31.2 ± 6.9. Hyponatraemia: 29.6 ± 6.5. Normonatraemia: 31.3 ± 6.9. *P* < .001	ASA		Anatomic TSA and reverse TSA
Martino et al 2024^58^	Cirrhosis associated with increased complications and health-care utilization following total shoulder arthroplasty.	Shoulder and Elbow	Retrospective cohort study	United States	Nationwide administrative database (exact database not specified in provided information)	1,254	Age: Mean 68 ± 8.4 yr for both groups *P* = .884	39% male both groups		Charlson-Deyo Comorbidity Index (CCI) Cirrhosis: Mean 6.1 ± 1.8 No cirrhosis: Mean 1.0 ± 1.2		Both anatomic and reverse TSA
Vanderham et al 2024^81^	Psychiatric comorbidities are an independent risk factor for perioperative and post-operative complications in patients undergoing primary total shoulder arthroplasty.	Journal of Shoulder and Elbow Surgery	Retrospective database analysis	United States	Nationwide administrative database	128,382	MHD: 68 ± 8.9 yr No MHD: 71 ± 8.9 yr *P* < .001 for both	MHD: 30% No MHD: 49% *P* < .001 for both		Charlson–Deyo Index		Primary TSA - both anatomic and reverse
Schairer et al 2015^78^	National utilization of reverse total shoulder arthroplasty in the United States.	Journal of Shoulder and Elbow Surgery	Retrospective national database analysis	United States	National Inpatient Sample (NIS), HCUP	66,485	rTSA: 72.7 ± 16.1 TSA: 67.4 ± 15.1 Hemi: 66.8 ± 28.1	rTSA: 36.4%; TSA: 49.6%; Hemi: 37.0%		Number of comorbidities - Mean: rTSA: 2.18; TSA: 1.86; Hemi: 2.21	Osteoarthritis 93.4% (n = 27,420) Avascular necrosis 2.0% (n = 594) Inflammatory arthritis 1.2% (n = 356) Proximal humerus fracture 1.2% (n = 349) Rotator cuff tear 1.0% (n = 306) Revision arthroplasty 0.5% (n = 155) Malunion/nonunion 0.4% (n = 116) Instability 0.1% (n = 41) Malignancy 0.0% (n = 14) Osteomyelitis 0.0% (n = 5)	Anatomic TSA
Polisetty et al 2021^73^	Determining the validity of the Outpatient Arthroplasty Risk Assessment (OARA) tool for identifying patients for safe same-day discharge after primary shoulder arthroplasty.	JSES	Retrospective review	United States	Single-surgeon institutional shoulder/elbow registry	422	71.6 ± 8.3 yr	48.30%	28.5 ± 6.0 kg/m^2^	OARA score	Primary elective shoulder arthroplasty	Anatomic and reverse TSA
Kishan et al 2025^44^	Perioperative complications and length of stay in patients with diabetes mellitus after aseptic revision total shoulder arthroplasty.	Journal of Orthopedic Reports	Retrospective cohort study	United States	ACS-NSQIP database	1,979	Age: Median 69 yr (IQR 62-75) overall No DM: 69 (62-75) DM: 69 (63-74) *P* = .99 (Wilcoxon rank sum test)	% male: Overall: 45.7% No DM: 44.4% DM: 50.7% *P* = .03 (Fisher's exact test)	BMI (mean ± SD): Overall: 31 ± 6.8 No DM: 30 ± 6.6 DM: 34 ± 6.9 *P* < .001 (one-way analysis of variance)	Comorbidity indices: ASA I: 1.2% ASA II: 36.0% ASA III: 58.8% ASA IV: 4.04% *P* = .22 (Pearson's chi-squared test) Hypertension: Overall: 1,310 (66.2%) No DM: 958 (60.8%) DM: 352 (87.1%) *P* < .001 (Fisher's exact test) Current smoker: Overall: 235 (11.9%) No DM: 189 (12.0%) DM: 46 (11.4%) *P* = .80 (Fisher's exact test) History of severe COPD: Overall: 155 (7.8%) No DM: 114 (7.2%) DM: 41 (10.2%) *P* = .06 (Fisher's exact test) Congestive heart failure: Overall: 22 (1.1%) No DM: 15 (1.0%) DM: 7 (1.7%) *P* = .19 (Fisher's exact test) Bleeding disorder: Overall: 61 (3.1%) No DM: 41 (2.6%) DM: 20 (4.95%) *P* = .02 (Fisher's exact test) Hematocrit <35%: Overall: 269 (13.6%) No DM: 190 (12.1%) DM: 79 (19.6%) *P* < .001 (Fisher's exact test) Total operation time (minutes, mean ± SD): Overall: 126.1 ± 62.4 No DM: 126.1 ± 62.4 DM: 125.8 ± 62.4 *P* = .93 (one-way analysis of variance)	Aseptic revision TSA	Revision total shoulder arthroplasty

*LOS*, length of stay; *ASA*, American Society of Anesthesiologists; *BMI*, body mass index; *IQR*, interquartile range; *SD*, standard deviation; *IDDM*, insulin-dependent diabetes mellitus; *mFI*, modified frailty index; *CKD*, chronic kidney disease; *COPD*, chronic obstructive pulmonary disease; *TXA*, tranexamic acid; *CCI*, Charlson Comorbidity Index; *TSA*, total shoulder arthroplasty; *rTSA*, reverse total shoulder arthroplasty; *aTSA*, anatomic total shoulder arthroplasty; *DM*, diabetes mellitus; *CHF*, congestive heart failure; *SDD*, same-day discharge; *MetS*, metabolic syndrome; *ICD-9/10*, International Classification of Diseases, Ninth and Tenth Revisions; *NIDDM*, non-insulin-dependent diabetes mellitus; *RA*, rheumatoid arthritis; *Htn*, hypertension; *GLP-1*, glucagon-like peptide-1; *FMS*, Fibromyalgia syndrome (from Moore et al 2024); *OHS*, overnight hospital stay (defined as >1 day; from Miltenberg et al 2024); *SHS*, short hospital stay (defined as 0–1 days; from Miltenberg et al 2024)..

**Table II:** Study summaries.

Author (yr)	Title	Aims/methods	Conclusion
Liu et al 2025^50^	Dehydration independently predicts prolonged hospital stay following aseptic revision total shoulder arthroplasty.	Retrospective review of a national surgical database assessing whether pre-operative dehydration, defined by elevated BUN/creatinine ratio, independently predicts early post-operative complications after aseptic revision total shoulder arthroplasty, using multivariable logistic regression for risk adjustment.	Pre-operative dehydration was associated with increased early post-operative morbidity and independently predicted prolonged hospitalization, indicating that hydration status may serve as a practical marker for perioperative risk stratification in revision shoulder arthroplasty.
Nazzal et al 2025^67^	Predictors of failing same-day discharge after shoulder arthroplasty: developing a model to improve outcomes and reduce health care cost.	Retrospective cohort study of consecutive shoulder arthroplasty cases intended for same-day discharge, evaluating demographic, clinical, and perioperative factors associated with failure of same-day discharge through multivariable logistic regression to develop a predictive model for post-operative hospital admission.	Older age, rotator cuff arthropathy, female sex, pre-operative opioid use, and prolonged anesthesia duration significantly increased the likelihood of failing same-day discharge. These findings support risk-stratified selection of candidates for outpatient arthroplasty and highlight the importance of coordinated perioperative planning to optimize resource utilization.
Areti et al 2025^4^	Impact of morbid obesity on post-operative outcomes in reverse total shoulder arthroplasty: A national inpatient sample analysis.	Retrospective cohort analysis of a national inpatient database evaluating whether morbid obesity is associated with adverse perioperative outcomes following reverse total shoulder arthroplasty, using comparative statistics and multivariate logistic regression to assess differences in complications, discharge status, and length of stay.	Morbid obesity was associated with higher complication rates, prolonged hospitalization, and increased likelihood of non-routine discharge after reverse total shoulder arthroplasty, highlighting the need for tailored perioperative optimization and post-operative management in this high-risk population.
Mayfield et al 2025^60^	Perioperative opioid use in total shoulder arthroplasty is associated with dose-dependent risk of major surgical and medical complications.	Retrospective analysis of a large national hospital database assessing whether perioperative opioid consumption, quantified in morphine milligram equivalents and stratified by quintiles, is associated with early surgical and medical complications after primary total shoulder arthroplasty.	Higher perioperative opioid exposure demonstrated a dose-dependent increase in post-operative complications, including infection, mechanical failure, and serious medical events, indicating that opioid requirements may serve as a meaningful predictor for risk stratification and perioperative optimization in total shoulder arthroplasty.
Liu et al 2024^54^	Severe malnutrition predicts early post-operative complications in aseptic revision total shoulder arthroplasty.	Retrospective national database study assessing whether pre-operative nutritional risk, quantified by the Geriatric Nutritional Risk Index and stratified by severity, independently predicts 30-d complications after revision total shoulder arthroplasty using multivariable logistic regression.	Severe malnutrition according to the GNRI significantly increased the risk of early post-operative complications, whereas moderate malnutrition did not, highlighting the GNRI as a practical tool for perioperative risk stratification in revision shoulder arthroplasty.
Mayfield et al 2025^61^	Tranexamic acid use in total shoulder arthroplasty continues to increase and is safe in high-risk patients.	Retrospective analysis of a national hospital database evaluating temporal trends in tranexamic acid use during primary total shoulder arthroplasty and assessing its association with transfusion requirements and thromboembolic complications, including a propensity-matched comparison and a subanalysis of high-risk patients with prior venous thromboembolism.	Expanded use of tranexamic acid was associated with reduced transfusion rates without increasing thromboembolic risk, even in patients with a history of venous thromboembolism, supporting its safety for broader perioperative use in shoulder arthroplasty
Liu et al 2024^49^	The association of hyponatremia and early post-operative complications in aseptic revision total shoulder arthroplasty.	Retrospective review of a national surgical database assessing whether pre-operative hyponatremia independently predicts 30-d complications after aseptic revision total shoulder arthroplasty, using multivariable logistic regression to compare outcomes with patients presenting normal sodium levels.	Hyponatremia was associated with increased early complications, including transfusion, reoperation, and prolonged hospitalization, supporting serum sodium as a practical marker for perioperative risk stratification in revision shoulder arthroplasty
Liu et al 2024^51^	Hypoalbuminemia predicts early post-operative complications following noninfectious revision total shoulder arthroplasty.	Retrospective national database review evaluating whether pre-operative hypoalbuminemia independently predicts 30-d complications after noninfectious revision total shoulder arthroplasty, using multivariable logistic regression to compare outcomes between patients with normal and reduced albumin levels	Hypoalbuminemia significantly increased the risk of early post-operative morbidity, including infection, transfusion, readmission, and prolonged hospitalization, highlighting serum albumin as a useful marker for perioperative risk stratification in revision shoulder arthroplasty
Liu et al 2024^53^	Elevated alkaline phosphatase independently predicts early post-operative complications in noninfectious revision total shoulder arthroplasty.	Retrospective national database analysis evaluating whether elevated pre-operative alkaline phosphatase predicts 30-d complications after noninfectious revision total shoulder arthroplasty, using multivariable logistic regression to compare outcomes with patients who had normal levels	Elevated alkaline phosphatase significantly increased the risk of early post-operative morbidity, including infection, transfusion, readmission, and prolonged hospitalization, indicating its value as a potential marker for perioperative risk stratification in revision shoulder arthroplasty
Liu et al 2024^52^	The geriatric nutritional risk index as a strong predictor of adverse outcomes following total shoulder arthroplasty.	Retrospective national database study evaluating whether malnutrition severity, quantified by the Geriatric Nutritional Risk Index and stratified into normal, moderate, and severe risk categories, independently predicts 30-d complications after total shoulder arthroplasty using multivariable logistic regression	Malnutrition identified by the Geriatric Nutritional Risk Index was a strong independent predictor of post-operative morbidity and mortality, with complication risk increasing proportionally with nutritional deficit, supporting pre-operative nutritional assessment for risk stratification in total shoulder arthroplasty.
Fassler et al 2024^22^	Components of metabolic syndrome as significant risk factors for post-operative complications following total shoulder arthroplasty: hypertension, diabetes, and obesity.	Retrospective national database study assessing whether metabolic syndrome independently predicts 30-d complications after total shoulder arthroplasty, with multivariable logistic regression used to adjust for demographic and comorbidity differences between metabolic syndrome and control cohorts.	Metabolic syndrome significantly increased the risk of cardiopulmonary and renal complications, non-home discharge, and prolonged hospitalization, underscoring the importance of pre-operative medical optimization in this patient population undergoing total shoulder arthroplasty.
Burns et al 2024^11^	Chronic kidney disease increases cost of care and readmission risk after shoulder arthroplasty.	Retrospective single-surgeon cohort study comparing health care utilization and 90-d encounter charges after primary shoulder arthroplasty in patients with and without chronic kidney disease, using univariate and multivariable regression to evaluate length of stay, transfusion, emergency utilization, readmission, and total post-operative costs.	Chronic kidney disease was associated with greater resource utilization, including increased transfusion risk, longer hospitalization, higher rates of emergency visits and readmissions, and elevated post-operative charges, emphasizing the importance of pre-operative optimization in this high-risk population.
Brandner et al 2024^8^	Impact of opioid dependence on outcomes following total shoulder arthroplasty.	Retrospective national readmissions database analysis comparing outcomes of primary total shoulder arthroplasty in patients with and without opioid dependence, using multivariate regression to assess 180-d complications, revision, readmission, length of stay, discharge disposition, and hospitalization costs.	Pre-operative opioid dependence was associated with significantly increased post-operative complications, greater revision and readmission risk, and higher resource utilization, supporting targeted strategies to reduce opioid dependence prior to shoulder arthroplasty to improve outcomes and decrease costs
Gruson et al 2023^29^	Does Morbid Obesity (BMI ≥40 kg/m^2^) Impact Operative Time, Blood Loss, Length of Stay, or Complications Following Anatomic Total Shoulder Arthroplasty?	Retrospective review of consecutive anatomic total shoulder arthroplasty cases assessing the impact of obesity classification on operative time, blood loss, perioperative complications, and hospital length of stay, using correlation analyses and regression modeling to identify predictors of prolonged hospitalization.	Morbid obesity did not increase operative time, blood loss, or complication rates but independently predicted longer length of stay, indicating that elevated BMI primarily affects post-operative resource utilization rather than surgical complexity in anatomic shoulder arthroplasty.
Papalia et al 2023^69^	Inpatient charges, complication, and revision rates for shoulder arthroplasty in Parkinson disease: a regional database study.	Retrospective analysis of a statewide all-payer database comparing primary shoulder arthroplasty outcomes in patients with and without Parkinson disease, evaluating inpatient charges, complication and revision rates, and readmissions, with regression modeling to control for demographic and comorbidity differences	Parkinson disease was associated with higher post-operative complications, increased revision risk, and greater inpatient resource utilization, indicating the need for careful risk counseling and perioperative planning for patients undergoing shoulder arthroplasty with this condition
Diamond et al 2023^18^	How does depressive disorder impact outcomes in patients with glenohumeral osteoarthritis undergoing primary reverse shoulder arthroplasty?	Retrospective national claims database study comparing outcomes of primary reverse shoulder arthroplasty in patients with and without depressive disorders, evaluating length of stay, 90-d medical complications, emergency department utilization, and episode-of-care costs, with sub-group analysis accounting for pre-operative emergency utilization	Depressive disorders were associated with longer hospitalization, higher medical complication rates, increased emergency department use, and greater care costs, indicating that mental health status influences post-operative outcomes and resource utilization after reverse shoulder arthroplasty.
Polisetty et al 2023^72^	Iron deficiency anemia is associated with increased medical and implant-related complications and length of stay for patients undergoing total shoulder arthroplasty.	Retrospective Medicare database review comparing outcomes of primary total shoulder arthroplasty in patients with and without iron deficiency anemia, using matched cohorts and multivariate logistic regression to evaluate effects on length of stay and 90-d medical and implant-related complications	Iron deficiency anemia was associated with longer hospitalization and substantially higher risks of medical and implant-related complications, including markedly increased transfusion and infection rates, underscoring the importance of pre-operative evaluation and optimization in this population
Puzzitiello et al 2022^75^	Patients with limited health literacy have worse pre-operative function and pain control and experience prolonged hospitalizations following shoulder arthroplasty.	Retrospective single-surgeon cohort study evaluating whether limited health literacy, identified pre-operatively using a validated screening tool, influences pre-operative symptom burden and perioperative outcomes after elective shoulder arthroplasty, with multivariable regression used to assess its effect on length of stay.	Limited health literacy was common and associated with worse pre-operative symptom severity and independently predicted longer hospitalization, indicating its relevance in perioperative planning and post-operative disposition after shoulder arthroplasty
Burns et al 2022^10^	Modifiable risk factors increase length of stay and 90-d cost of care after shoulder arthroplasty.	Retrospective review across a regional health system assessing the impact of predefined modifiable risk factors—including anemia, malnutrition, obesity, uncontrolled diabetes, tobacco use, and opioid use—on 90-d encounter charges, hospital length of stay, emergency department utilization, and readmissions following shoulder arthroplasty, using multivariable modeling to evaluate cost and resource use.	Malnutrition, obesity, and anemia significantly increased post-operative costs and resource utilization, particularly length of stay and readmissions, supporting targeted pre-operative optimization programs to reduce financial burden and improve outcomes in shoulder arthroplasty.
Doan et al 2021^19^	Increasing severity of anemia is associated with poorer 30-d outcomes for total shoulder arthroplasty.	Retrospective national surgical database analysis evaluating whether increasing pre-operative anemia severity, stratified by hematocrit levels, independently influences post-operative outcomes after primary total shoulder arthroplasty, using multivariable logistic regression to assess complications, readmissions, reoperations, and length of stay.	Greater anemia severity was progressively associated with higher complication, readmission, reoperation, and prolonged hospitalization rates, indicating hematocrit as a practical tool for pre-operative risk stratification and optimization in total shoulder arthroplasty.
Flamant et al 2021^23^	Malnutrition in elective shoulder arthroplasty: a multi-institutional retrospective study of pre-operative albumin and adverse outcomes.	Retrospective review of primary and revision shoulder arthroplasty at two tertiary centers assessing whether low pre-operative albumin levels predict extended hospitalization, 90-d readmission, and discharge to postacute care facilities, using multivariable analysis to adjust for demographic and clinical risk factors.	Malnutrition, identified by low albumin, was more common in revision cases and independently increased the risk of prolonged hospitalization, with a possible greater need for postacute care, supporting albumin assessment for perioperative risk stratification in shoulder arthroplasty.
Wang et al 2021^82^	Increased severity of anemia is associated with post-operative complications following primary total shoulder arthroplasty.	Retrospective national surgical database study evaluating whether the severity of pre-operative anemia, stratified by WHO hematocrit thresholds, independently predicts 30-d complications after primary total shoulder arthroplasty using multivariable logistic regression.	Increasing anemia severity was associated with a stepwise rise in post-operative morbidity, including transfusion, cardiopulmonary events, reoperation, prolonged hospitalization, and mortality, supporting medical optimization of patients with moderate to severe anemia prior to shoulder arthroplasty.
Lunati et al 2021^57^	Pre-operative depression is a risk factor for complication and increased health care utilization following total shoulder arthroplasty.	Retrospective administrative database analysis comparing outcomes of primary total shoulder arthroplasty in patients with and without pre-operative depression, using multivariate adjustment to evaluate post-operative complications, prosthetic failures, and health-care utilization within one year.	Pre-operative depression was common and independently associated with higher complication rates, increased prosthetic and wound-related failures, and greater health-care utilization, indicating that mental health status may influence post-operative risk and warrants consideration during pre-operative planning.
Wilson et al 2021^84^	Pre-operative opioid use is an independent risk factor for complication, revision, and increased health care utilization following primary total shoulder arthroplasty.	Retrospective administrative claims analysis evaluating the dose-dependent relationship between pre-operative opioid use and post-operative outcomes after primary total shoulder arthroplasty, stratifying patients by average daily morphine equivalents over the 6 mo prior to surgery and using multivariate regression to assess complications, resource utilization, and revisions.	Higher pre-operative opioid exposure was common and demonstrated a dose-dependent increase in complications, health care utilization, revision risk, and costs, supporting opioid reduction prior to shoulder arthroplasty as a modifiable target for optimization
Dacombe et al 2020^16^	Predictors of length of stay following shoulder arthroplasty in a high-volume UK center.	Retrospective review of shoulder arthroplasty cases at a high-volume UK center evaluating patient, comorbidity, and perioperative factors associated with inpatient length of stay, with multivariable linear regression used to identify independent predictors of prolonged hospitalization.	Advanced age, female sex, cardiac and renal comorbidities, intraoperative complications, and transfusion independently increased length of stay, supporting targeted optimization and reduction of perioperative complications to improve inpatient efficiency following shoulder arthroplasty.
Menendez et al 2019^62^	Delayed hospital discharge after total shoulder arthroplasty: why, and who is at risk?	Retrospective review of elective primary total shoulder arthroplasty assessing reasons for extended hospitalization and identifying pre-operative factors associated with prolonged length of stay, using manual chart review to classify causes and multivariable logistic regression to determine independent predictors	Most extended hospitalizations were due to pain, minor medical issues, or limited social support rather than surgical complications, with sociodemographic and psychological factors emerging as key predictors, supporting comprehensive perioperative management beyond technical care alone.
Biron et al 2020^7^	A Novel Machine Learning Model Developed to Assist in Patient Selection for Outpatient Total Shoulder Arthroplasty.	Retrospective analysis of a national surgical database using a random forest machine learning model to predict short-stay outpatient candidacy after elective total shoulder arthroplasty, followed by multivariable logistic regression to identify demographic and comorbidity factors associated with length of stay.	Machine learning accurately identified patients likely to achieve short stays, with younger age and male sex favoring outpatient eligibility, while cardiopulmonary comorbidities and higher ASA classification predicted longer hospitalization, supporting its potential role in outpatient candidate selection.
Anthony et al 2019^3^	Utilization and Real-world Effectiveness of Tranexamic Use in Shoulder Arthroplasty: A Population-based Study.	Retrospective national claims analysis evaluating trends in tranexamic acid use during shoulder arthroplasty and its association with transfusion risk, post-operative complications, and hospital resource utilization, using multilevel multivariable regression to control for patient and hospital factors.	Tranexamic acid use was low but increased over time and was associated with markedly reduced transfusion risk and lower complication rates without added cost or safety concerns, supporting its broader adoption in shoulder arthroplasty
Lee et al 2019^48^	Low Serum Albumin Levels are Associated with Increased 30-d Cardiopulmonary Complications, Reoperation, and Readmission Rates Following Total Shoulder Arthroplasty.	Propensity-matched NSQIP analysis evaluating hypoalbuminemia as a predictor of 30-d morbidity after primary TSA. Patients with low pre-operative serum albumin were compared with matched controls for acute post-operative complications using multivariate logistic regression.	Hypoalbuminemia was strongly associated with higher pulmonary, cardiac, thromboembolic, infectious, and hematologic complications, along with greater reoperation, readmission, and prolonged hospitalization. Low albumin independently increased risk for pulmonary events, transfusion, reoperation, and readmission, supporting its value in pre-operative risk stratification and optimization before TSA.
Kim et al 2018^42^	Predicting adverse events, length of stay, and discharge disposition following shoulder arthroplasty: a comparison of the Elixhauser Comorbidity Measure and Charlson Comorbidity Index.	Queried a national inpatient database to evaluate whether the Elixhauser Comorbidity Measure provides superior predictive accuracy to the Charlson Comorbidity Index for forecasting adverse events, resource utilization, and discharge disposition following total shoulder and reverse total shoulder arthroplasty using logistic regression models and concordance statistics.	A model incorporating the Elixhauser Comorbidity Measure demonstrated superior discrimination for mortality, complications, extended hospitalization, and nonroutine discharge, indicating its value as a more reliable tool for guiding perioperative care pathways and supporting value-based reimbursement strategies in shoulder arthroplasty.
Izquierdo-Fernández et al 2018^37^	Reverse shoulder arthroplasty in obese patients: analysis of functionality in the medium-term.	Evaluated functional and radiographic outcomes of reverse shoulder arthroplasty in patients stratified by body mass index, comparing two BMI-based sub-group analyses and assessing complications, length of stay, radiographic changes, and standardized functional scores.	Patients with BMI ≥35 demonstrated significantly poorer functional results, while complication rates and radiographic findings were similar across BMI groups, indicating that severe obesity adversely affects post-operative function without increasing measurable implant-related complications.
Fu et al 2017^25^	The impact of insulin dependence on short-term post-operative complications in diabetic patients undergoing total shoulder arthroplasty.	The study analyzed a national surgical database to compare 30-d post-operative complications, readmissions, and length of stay across non-diabetic, non–insulin-dependent diabetic, and insulin-dependent diabetic patients undergoing total shoulder arthroplasty. Multivariable logistic regression was used to determine the independent effect of insulin dependence on post-operative outcomes after adjusting for demographic and comorbidity factors.	Non–insulin-dependent diabetes mellitus did not confer additional post-operative risk after adjustment, whereas insulin dependence independently predicted higher rates of several complications. Insulin status should be incorporated into pre-operative risk stratification for diabetic patients undergoing shoulder arthroplasty.
Issa et al 2016^36^	Total Shoulder Arthroplasty Demographics, Incidence, and Complications—A Nationwide Inpatient Sample Database Study.	Evaluated contemporary national trends in total shoulder arthroplasty using the Nationwide Inpatient Sample (1998-2010), assessing temporal changes in utilization, demographic patterns, and inpatient outcomes. Multivariable regression was applied to determine independent associations between patient factors, complications, and length of stay.	Incidence of total shoulder arthroplasty increased substantially over the study period, while complication rates remained stable and length of stay declined. Older age, female sex, and greater comorbidity burden were independently associated with higher complication risk, and several demographic and insurance factors contributed to prolonged hospitalization.
Weinreb et al 2016^83^	Shoulder Arthroplasty: Disposition and Perioperative Outcomes in Patients With and Without Rheumatoid Arthritis.	Retrospective analysis of a national inpatient database comparing perioperative complication profiles and hospital disposition between patients undergoing shoulder arthroplasty with and without rheumatoid arthritis, using multivariable regression to adjust for demographic and hospital factors.	Rheumatoid arthritis was associated with increased transfusion requirements and modest differences in perioperative resource use and discharge patterns, while overall complication rates remained comparable. Further work is needed to clarify drivers of these disparities and optimize perioperative management.
Mollon et al 2016^64^	The influence of a history of clinical depression on peri-operative outcomes in elective total shoulder arthroplasty: a 10-yr national analysis.	This study used a national inpatient database to evaluate whether a documented history of depression independently influences peri-operative complications and discharge disposition in patients undergoing elective total shoulder arthroplasty. Multivariable analyses were performed to identify associations between depression and adverse in-hospital outcomes.	Depression was independently associated with a higher risk of several post-operative complications and non-home discharge. The findings support routine pre-operative screening and targeted peri-operative management to mitigate risk in this vulnerable patient group.
Kandil et al 2016^39^	Blood transfusion after total shoulder arthroplasty: Which patients are at high risk?	Used a national inpatient database to evaluate contemporary transfusion incidence in TSA and to identify pre-operative variables independently associated with transfusion. Assessed relationships between transfusion status and hospital utilization metrics.	Several pre-operative comorbidities were independently associated with transfusion, allowing clearer stratification of higher-risk patients. Transfusion was also linked to greater resource use, underscoring the importance of targeted risk counseling and perioperative optimization.
Murphy et al 2016^66^	Metabolic syndrome and shoulder arthroplasty: epidemiology and peri-operative outcomes.	Retrospective analysis of national inpatient discharge data to evaluate temporal trends in metabolic syndrome among shoulder arthroplasty patients and to examine its independent association with peri-operative complications, length of stay, and discharge disposition using multivariable logistic regression.	Metabolic syndrome demonstrated a marked rise over the study period and independently increased the risk of peri-operative morbidity and resource utilization. Enhanced recognition and peri-operative optimization of metabolic syndrome may improve outcomes in shoulder arthroplasty.
Sivasundaram et al 2016^79^	Pre-operative risk factors for discharge to a postacute care facility after shoulder arthroplasty.	Using a national inpatient database, the study evaluated patient and hospital characteristics associated with discharge to postacute care following total or reverse shoulder arthroplasty. Multivariate modeling was applied to identify independent predictors while adjusting for relevant covariates.	The analysis identified several demographic, clinical, and procedural factors that independently increase the likelihood of postacute care discharge, providing a basis for pre-operative risk stratification and more efficient discharge planning.
Garcia et al 2016^26^	Malnutrition: a marker for increased complications, mortality, and length of stay after total shoulder arthroplasty.	Retrospective cohort study using a national surgical quality database to quantify malnutrition prevalence in TSA and evaluate post-operative complication risk associated with low pre-operative albumin. Malnutrition was defined biochemically, and outcomes were compared between nutritional groups using propensity-adjusted analyses.	Pre-operative hypoalbuminemia was associated with substantially increased post-operative morbidity and early mortality in TSA. Malnutrition independently predicted higher transfusion requirements, prolonged hospitalization, and markedly elevated short-term mortality risk.
Basques et al 2015^5^	Length of Stay and Readmission After Total Shoulder Arthroplasty: An Analysis of 1505 Cases.	The study examined patient-related predictors of extended hospital stay and 30-d readmission following primary total shoulder arthroplasty using a national surgical quality database. Bivariate and multivariate regression models were applied to evaluate associations between pre-operative characteristics and post-operative utilization outcomes.	Specific comorbidities were independently linked to prolonged hospitalization and early readmission, highlighting the value of targeted risk stratification to guide pre-operative counseling and post-operative planning.
Matsen et al 2015^59^	Factors Affecting Length of Stay, Readmission, and Revision After Shoulder Arthroplasty: A Population-Based Study.	Analyzed statewide administrative data from primary shoulder arthroplasty procedures to identify patient, diagnosis, and provider factors independently associated with prolonged hospitalization, 90-d readmission, and revision surgery. Multivariable models were used to determine which characteristics predicted each adverse outcome.	Distinct clinical and demographic variables were linked to extended stay, early readmission, and need for revision. Readily identifiable patient and provider characteristics may inform perioperative optimization strategies and support targeted efforts to reduce complications and associated health-care costs.
Pappou et al 2014^70^	Outcomes and Costs of Reverse Shoulder Arthroplasty in the Morbidly Obese: A Case Control Study.	The study compared post-operative outcomes, complications, discharge disposition, and hospital costs between morbidly obese patients and matched non-obese controls undergoing primary reverse shoulder arthroplasty. A joint registry cohort with minimum 2-yr follow-up was analyzed using standardized functional measures, comorbidity indices, perioperative metrics, and recorded post-operative events.	Reverse shoulder arthroplasty demonstrated comparable functional gains and complication rates in morbidly obese patients relative to non-obese controls, though obesity was associated with increased perioperative resource utilization, higher costs, and a greater likelihood of requiring post-acute rehabilitation rather than home discharge.
Ponce et al 2014^74^	Diabetes as a risk factor for poorer early post-operative outcomes after shoulder arthroplasty.	The study analyzed a national inpatient database to compare post-operative mortality, complications, length of stay, and discharge disposition between diabetic and non-diabetic patients undergoing shoulder arthroplasty, using bivariate and multivariable logistic regression to determine independent associations.	Pre-existing diabetes independently increased the risk of perioperative morbidity, mortality, prolonged hospitalization, and non-home discharge after shoulder arthroplasty, without influencing hospital costs. Further prospective investigation is warranted.
Griffin et al 2014^27^	Morbid obesity in total shoulder arthroplasty: risk, outcomes, and cost analysis.	Retrospective analysis of a national inpatient database comparing post-operative complications, mortality, and resource utilization between morbidly obese and non-obese patients undergoing total shoulder arthroplasty using multivariate logistic regression modeling.	Morbid obesity was associated with greater post-operative respiratory complications, longer hospitalization, and higher inpatient costs, while most other complication rates and early mortality showed no significant increase. Further investigation is warranted to refine risk stratification and support pre-operative counseling.
Johnson et al 2014^38^	Does pre-operative American Society of Anesthesiologists score relate to complications after total shoulder arthroplasty?	Retrospective cohort analysis evaluating whether ASA classification predicts surgical complications, medical complications, and hospitalization duration following primary and revision total shoulder arthroplasty. Logistic regression was used to model complication risk as a function of ASA score.	Higher ASA class was independently associated with increased surgical complications, including prosthesis-related failure, and with longer hospital stay, but showed no association with medical complications. The ASA score may serve as a useful tool for perioperative risk stratification and counseling.
Griffin et al 2013^28^	Obstructive sleep apnea as a risk factor after shoulder arthroplasty.	Leveraged a national inpatient database to compare post-operative in-hospital complications, resource use, and length of stay between shoulder arthroplasty patients with and without a diagnosis of obstructive sleep apnea using multivariate logistic regression.	OSA was not associated with higher in-hospital morbidity, mortality, or costs and was linked to a modestly shorter hospital stay, indicating it may not represent a significant perioperative risk factor in shoulder arthroplasty.
Humphries et al 2008^35^	Effect of the Deyo score on outcomes and costs in shoulder arthroplasty patients.	Evaluated whether pre-operative comorbidity burden, quantified by the Deyo index, independently predicted perioperative mortality, complications, hospital stay, and costs in shoulder arthroplasty. Outcomes were compared across comorbidity strata using chi-square and F-tests, with multivariable logistic and linear regression models adjusting for key demographic and provider factors.	Greater comorbidity burden was independently associated with increased perioperative risk and higher resource utilization. The Deyo index functioned as a meaningful predictor to inform patient selection and risk–benefit discussions for shoulder arthroplasty.
Elsabbagh et al 2025^21^	Does use of glucagon-like peptide-1 agonists increase perioperative complications in patients undergoing shoulder arthroplasty?	Retrospective cohort study using a national database to compare post-operative and joint-related outcomes in T2DM patients undergoing primary TSA with or without GLP-1 therapy. Propensity-score matching and multivariable logistic regression were used to evaluate 90-d medical complications, length of stay, and 2-yr joint-related events.	GLP-1 therapy was neither associated with increased post-operative medical or joint-related complications nor with prolonged length of stay. Findings suggest perioperative GLP-1 use does not elevate surgical risk in TSA and may be safely continued, supporting further prospective evaluation.
Alder et al 2024^2^	Increasing severity of pre-operative anemia is associated with higher post-operative medical and surgical complications after primary shoulder arthroplasty.	Evaluated midterm outcomes of primary shoulder arthroplasty stratified by pre-operative anemia severity using a single-institution registry with ≥2-yr follow-up. Compared medical and surgical complications, reoperations, revisions, and implant survivorship across anemia categories.	Greater pre-operative anemia severity was associated with higher rates of medical and surgical complications. Post-operative transfusion further increased complication rates and short-term mortality, underscoring the need for targeted perioperative optimization and monitoring in anemic patients.
Khokhar et al 2024^40^	Does morbid obesity negatively impact perioperative outcomes following elective reverse shoulder arthroplasty?: a propensity-matched comparative study.	Retrospective propensity-matched analysis of primary elective rTSA cases using a uniform short-stem implant system to evaluate the independent association of morbid obesity with perioperative surgical and medical outcomes. Multivariable regression assessed relationships between obesity status and operative parameters, post-operative complications, disposition, and short-term unplanned health care utilization.	Morbid obesity did not independently worsen perioperative or short-term post-operative outcomes. With appropriate pre-operative optimization, morbid obesity alone should not be considered a contraindication to elective rTSA.
Destine et al 2024^17^	Effect of hospital length of stay on functional outcomes and readmission after total shoulder arthroplasty.	A retrospective cohort study evaluated whether post-operative length of stay (≤24 vs. > 24 h) was associated with readmissions, revisions, complications, or functional outcomes following primary total shoulder arthroplasty. Demographic, perioperative, comorbidity, and discharge variables were analyzed to identify factors predictive of prolonged hospitalization.	Prolonged post-operative stay was associated with higher comorbidity burden and increased likelihood of discharge to a nonhome setting but did not influence complication rates, readmissions, revisions, or patient-reported outcomes.
Moore et al 2024^65^	Fibromyalgia syndrome is an independent predictor of increased complications, revision, and cost of admission within 180 d of total shoulder arthroplasty.	Retrospective analysis of a national readmissions database to compare post-operative complication profiles between patients with and without fibromyalgia undergoing primary elective TSA. Cohorts were matched 1:1 on key demographic and comorbidity variables, and multivariate modeling was used to isolate the independent effect of fibromyalgia on post-operative outcomes and health-care utilization.	Fibromyalgia independently increased the likelihood of both medical and surgical complications after TSA and was associated with higher revision rates and greater admission costs. Awareness of this elevated risk profile is warranted when planning and counseling patients undergoing TSA.
Miltenberg et al 2024^63^	Prediction of overnight stay following shoulder arthroplasty utilizing machine learning.	This study evaluated multiple machine learning models to predict likelihood of overnight hospital stay after primary elective total shoulder arthroplasty using a national surgical outcomes database. Models were trained and validated against post-operative disposition categories, with performance compared using discrimination, calibration, and decision curve analyses.	Machine learning—particularly artificial neural networks—provided superior predictive accuracy over traditional regression approaches for forecasting need for post-operative admission. These models can support more precise outpatient selection, inform pre-operative counseling, and facilitate resource optimization.
Kopriva et al 2024^45^	Tramadol use before total shoulder arthroplasty: patients have lower risk of complications and resource utilization than those using traditional opioids.	Retrospective cohort analysis of national claims data to compare post-operative resource utilization, complications, and revision risk in TSA patients stratified by pre-operative analgesic exposure (opioid-naïve, tramadol, traditional opioids, or combination). Multivariate modeling adjusted for baseline differences and evaluated temporal prescribing trends.	Pre-operative tramadol use was associated with a post-operative risk profile similar to opioid-naïve patients, whereas traditional opioid use conferred increased complications, higher resource utilization, and elevated revision risk. Findings support tramadol as a comparatively lower-risk alternative to traditional opioids in the pre-operative TSA setting.
Kim et al 2024^43^	Cumulative effect of chronic dehydration and age on post-operative complications after total shoulder arthroplasty.	This study assessed the effect of pre-operative dehydration and age on 30-d complications after total shoulder arthroplasty using ACS-NSQIP data (2015-2019) with multivariable regression analysis.	Pre-operative dehydration, particularly in elderly patients, was independently associated with higher complication rates, longer hospital stay, and increased nonhome discharge following total shoulder arthroplasty.
Hartwell et al 2019^31^	Total shoulder arthroplasty: Risk factors for a prolonged length of stay. A retrospective cohort study.	The study used a national surgical quality database to compare patients undergoing total shoulder arthroplasty by post-operative length of stay (≤24 h vs. > 24 h). Univariate and multivariate analyses evaluated pre-operative comorbidities, demographic characteristics, and intraoperative variables associated with extended hospitalization.	Several medical comorbidities and higher perioperative risk profiles were independently associated with hospital stays exceeding 24 h, whereas male sex was protective. These findings highlight key patient and operative factors that may inform perioperative optimization and outpatient pathway selection.
Lonergan et al 2019^56^	Mood disorders do not predict a more difficult course after primary anatomic total shoulder arthroplasty.	Evaluated whether pre-existing mood disorders influence post-operative pain trajectories, narcotic consumption, length of stay, or early complications following primary anatomic TSA. Patients were stratified by documented depression/anxiety or use of mood-stabilizing medications, with serial pain scores, opioid requirements, and 90-d post-operative outcomes collected prospectively.	Mood disorders did not adversely affect early recovery after primary anatomic TSA. Post-operative pain, opioid use, length of stay, and complication profiles were comparable between groups, suggesting mood disorders are not a meaningful risk factor for early outcomes or bundled payment modeling in this setting.
Hammond et al 2003^30^	Surgeon Experience and Clinical and Economic Outcomes for Shoulder Arthroplasty.	Evaluated whether surgeon case volume was associated with clinical and economic outcomes following total shoulder arthroplasty or hemiarthroplasty, using a statewide hospital discharge database and multivariable adjustment for patient, procedural, and hospital factors.	Higher surgeon volume was associated with lower complication rates and shorter hospital stays, with no significant difference in adjusted costs. Findings support the value of experience and ongoing training in optimizing shoulder arthroplasty outcomes.
Lee et al 2020^47^	Pre-operative hyponatremia is associated with post-operative major morbidity, prolonged length of stay, non-home discharge, and readmission in total shoulder arthroplasty.	This study used a national surgical quality database to evaluate whether pre-operative hyponatraemia independently predicts adverse 30-d outcomes following anatomic or reverse total shoulder arthroplasty. Multivariable logistic regression analyses were performed with stringent statistical correction to assess associations with post-operative morbidity and health-care utilization metrics.	Pre-operative hyponatraemia was an independent predictor of increased short-term morbidity and higher post-operative resource utilization after total shoulder arthroplasty. The authors highlight the need for thorough pre-operative optimization and call for further work to clarify the effect of hyponatraemia severity and correction on perioperative outcomes.
Martino et al 2024^58^	Cirrhosis associated with increased complications and health-care utilization following total shoulder arthroplasty.	Evaluated short-term post-operative outcomes of elective primary TSA in patients with cirrhosis using a nationwide database. Cirrhotic patients were matched 1:1 with non-cirrhotic controls, and post-operative complications and hospital utilization metrics were compared using adjusted statistical analyses.	Cirrhosis was associated with substantially increased medical and implant-related complications, higher post-operative risk overall, and greater resource utilization following TSA. Findings highlight the need for targeted pre-operative optimization and risk-mitigation strategies in this population.
Vanderham et al 2024^81^	Psychiatric comorbidities are an independent risk factor for perioperative and post-operative complications in patients undergoing primary total shoulder arthroplasty.	Evaluated the impact of pre-operative mental health diagnoses on perioperative adverse events, length of stay, and medical/mechanical complications within 180 d after primary TSA using a nationwide administrative database and multivariable logistic regression.	Pre-operative mental health disorders independently increased the likelihood of perioperative and mechanical complications and revision, indicating a need for heightened perioperative counseling and post-operative surveillance in this population.
Schairer et al 2015^78^	National utilization of reverse total shoulder arthroplasty in the United States.	Used a national inpatient database to characterize utilization patterns and surgical indications for hemiarthroplasty, TSA, and rTSA across the United States. Examined associated diagnoses and hospital factors, and applied multivariable regression to identify determinants of prolonged length of stay.	rTSA represented a substantial proportion of arthroplasty procedures, with notable uptake for proximal humerus fractures in addition to cuff-related pathology. Procedure choice varied by indication, and hospital stay was influenced by patient comorbidity and insurance status.
Polisetty et al 2021^73^	Determining the validity of the Outpatient Arthroplasty Risk Assessment (OARA) tool for identifying patients for safe same-day discharge after primary shoulder arthroplasty.	Retrospective single-surgeon study evaluating the utility of the Outpatient Arthroplasty Risk Assessment (OARA) score for determining appropriateness for same-day discharge after primary shoulder arthroplasty, comparing score thresholds and predictive performance against ASA-PS classification and prior lower-extremity arthroplasty criteria	An OARA cutoff of ≤110 points effectively and conservatively identified candidates for same-day discharge, with low emergency utilization and acceptable readmission rates, supporting its use as a screening tool for outpatient shoulder arthroplasty.
Kishan et al 2025^44^	Perioperative complications and length of stay in patients with diabetes mellitus after aseptic revision total shoulder arthroplasty.	Used a national surgical registry to evaluate whether diabetes mellitus independently influences perioperative complications and hospital length of stay following aseptic revision total shoulder arthroplasty. Applied multivariate logistic regression to identify clinical and perioperative predictors of major complications and prolonged admission.	Diabetes was not an independent determinant of short-term complications or extended hospital stay after aseptic revision TSA. Outcomes were more strongly associated with other comorbidities, highlighting the need for individualized risk stratification focusing on factors such as hypertension and low pre-operative hematocrit.

*ASA*, American Society of Anesthesiologists; *BMI*, body mass index; *BUN*, blood urea nitrogen; *TSA*, total shoulder arthroplasty; *rTSA*, reverse total shoulder arthroplasty; *GLP-1*, glucagon-like peptide-1; *VTE*, venous thromboembolism; *DD*, discharge disposition; *PAC*, post-acute care; *CNS*, central nervous system; *HA*, hospital admission; *MHD*, mental health disorder.

Indications included osteoarthritis, rotator cuff arthropathy, fractures, inflammatory disease, and revision procedures, with both anatomic and reverse arthroplasty represented.

### Risk of bias

Risk of bias in the 64 non-randomized studies was assessed using ROBINS-I (Fig. 2). Overall, 35.9% of studies were rated moderate and 64.1% serious risk of bias, with none rated low. Risk was primarily driven by confounding, missing data, and selective reporting.

#### Body mass index

The relationship between body mass index (BMI) and post-operative LOS was evaluated in 13 studies, with mixed findings.^4^^,^^7^^,^^10^^,^^17^^,^^27^^,^^29^^,^^31^^,^^37^^,^^40^^,^^47^^,^^62^^,^^70^^,^^75^ Morbid obesity (BMI ≥40 kg/m^2^) was most consistently associated with modestly increased LOS. Areti et al^4^ reported mean LOS of 2.23 ± 3.25 days in morbidly obese patients vs. 1.87 ± 1.89 days in non-morbidly obese patients (mean difference +0.36 days; *P* < .001). Griffin et al^27^ found LOS increased from 2.56 ± 2.0 days in non-obese patients to 2.84 ± 1.77 days in morbidly obese patients (*P* = .003), and Gruson et al^29^ reported a median LOS of 2 days for BMI <30 and 2 days (interquartile range 2-4) for BMI ≥40, with a significant incidence rate ratio for morbid obesity (incidence rate ratio 1.316; *P* = .038). Pappou et al^70^ also observed higher LOS in BMI ≥40 (3.1 ± 2.6 vs. 2.6 ± 1.3 days), though this was not statistically significant (*P* = .823).

For patients with a BMI between 30 and 40 kg/m^2^, any association with LOS was generally weak and often non-significant. Izquierdo-Fernández et al^37^ observed no significant differences for BMI ≥30 (5.83 ± 1.42 days) vs. <30 (5.18 ± 1.25 days; *P* = .222) or BMI ≥35 (6.00 ± 1.69 days) vs. <35 (5.43 ± 1.25 days; *P* = .327). Burns et al^10^ reported 2.4 ± 2.4 days in obese vs. 2.2 ± 1.6 days in non-obese patients (*P* = .158), and Khokhar et al^40^ found no difference in matched cohorts (median 2 [interquartile range 1-3] vs. 2 [2-3]; *P* = .654).

Several studies examined BMI as a continuous predictor, with variable results. Menendez et al^62^ reported a higher mean BMI in patients with prolonged LOS (32.6 ± 7.5 vs. 30.2 ± 5.8; *P* = .004), while Lee et al^47^ identified a non-significant effect per kg/m^2^ (odds ratio [OR] 1.01, 99% confidence interval [CI] 1.00-1.02). In contrast, Puzzitiello et al^75^ (β = 0.04; *P* = .54), Destine et al^17^ (OR 1.03, 95% CI 0.95-1.12; *P* = .413), Biron et al^7^ (31.7 vs. 31.2; *P* = .062), and Hartwell et al^31^ (31.1 vs. 31.0; *P* = .29) observed no significant associations. Additionally, Kishan et al^44^ examined BMI >30 as a predictor and found no significant association with LOS (OR 1.3, 95% CI 0.6-2.7).

Collectively, these findings suggest that morbid obesity (BMI ≥40) may modestly prolong LOS, whereas general obesity (BMI 30-40) shows inconsistent effects and BMI as a continuous variable exerts only a minimal or non-significant influence on post-operative LOS across arthroplasty populations.

## Comorbidities

### Diabetes

Diabetic status was consistently associated with prolonged hospital LOS in nine studies.^5^^,^^7^^,^^10^^,^^25^^,^^47^^,^^62^^,^^63^^,^^74^^,^^79^ Burns et al^10^ reported LOS of 2.8 ± 2.8 days in patients with uncontrolled diabetes vs. 2.3 ± 1.7 days in non-diabetic controls (*P* = .019). Fu et al^25^ demonstrated higher odds of extended LOS in insulin-dependent diabetes mellitus (OR 1.38, 95% CI 1.04-1.82; *P* = .025), while non–insulin-dependent diabetes mellitus was not significant (OR 1.13, 95% CI 0.92-1.39; *P* = .255). Basques et al^5^ reported a more pronounced effect (OR 2.37, 95% CI 1.53-3.66; *P* < .001), and Ponce et al^74^ observed longer LOS in complicated vs. uncomplicated diabetes (3.4 ± 2.8 vs. 2.7 ± 2.2 days; *P* < .001). Kishan et al^44^ further demonstrated that patients with diabetes undergoing aseptic revision shoulder arthroplasty had longer LOS than non-diabetic patients (1.98 ± 2.20 vs. 1.72 ± 1.82 days; *P* = .03), with higher odds of prolonged LOS (3.5% vs. 1.5%; *P* = .02). Other studies confirmed the association as follows: Menendez et al^62^ (28% vs. 9%; *P* < .001), Biron et al^7^ (25.8% vs. 16.3%; *P* < .001), Sivasundaram et al^79^ (OR 1.51, 95% CI 1.29-1.77; *P* < .001), Miltenberg et al^63^ (OR 2.85, 95% CI 1.88-3.72; *P* < .001), and Lee et al^47^ (OR 1.49, 99% CI 1.28-1.73).

### American Society of Anesthesiologists grade

American Society of Anesthesiologists (ASA) grade was also a strong predictor of LOS in seven studies.^7^^,^^38^^,^^62^^,^^63^^,^^67^^,^^73^^,^^75^ Johnson et al^38^ demonstrated mean LOS of 2.6 days for ASA 1-2 vs. 3.3 days for ASA 3-4 (effect size 0.46; *P* < .001). Menendez et al^62^ found 39% of patients with ASA ≥3 experienced prolonged LOS vs. 17% with ASA ≤2 (*P* < .001), and Biron et al^7^ reported 77.0% vs. 50.1% for long vs. short LOS in ASA >2 patients (*P* < .001). Miltenberg et al^63^ observed 88% increased odds of prolonged LOS with ASA 3-5 (OR 1.88, 95% CI 1.58-2.23; *P* < .001). Other studies showed trends without reaching significance.^67^^,^^73^^,^^75^

### Functional and frailty scores

Functional and frailty constructs were associated with stepwise increases in LOS in three studies.^33^^,^^35^^,^^36^ Humphries et al^35^ reported mean LOS of 3.2, 3.5, and 4.3 days for Deyo scores 0, 1-4, and 5-36, respectively (*P* < .001), with similar adjusted means. Issa et al^36^ demonstrated large effect sizes for Deyo scores ≥1 (2 vs. 0: +15.25, 95% CI 13.16-17.39; *P* < .0001). Frailty, measured by the modified frailty index (mFI), was also predictive: Holzgrefe et al^33^ reported mean LOS of 1.88 ± 2.9 days for mFI 0 vs. 2.43 ± 2.1 days for mFI ≥2 (*P* < .001), with similar trends in patients aged ≥70 years.

### Other comorbidities

Other comorbidities further influenced LOS mentioned in 10 studies.^11^^,^^16^^,^^22^^,^^41^^,^^47^^,^^58^^,^^59^^,^^66^^,^^69^^,^^78^ Charlson Comorbidity Index (CCI) and comorbidity counts were strong predictors: Schairer et al^78^ observed ORs for prolonged LOS rising from 1.69 for 2 comorbidities to 5.64 for ≥4 comorbidities. Kim et al^42^ reported prolonged LOS increased with Elixhauser Comorbidity Measure and CCI counts (*P* < .0001), with predictive model areas under the curve of 0.752 and 0.698, respectively. Matsen et al^59^ demonstrated CCI >1 associated with 1.73× LOS and elevated risk of prolonged LOS in total shoulder arthroplasty (85.7%) and HA (92.1%).

Disease-specific comorbidities were also relevant: chronic kidney disease (Burns et al^11^: 2.8 ± 1.3 vs. 2.3 ± 1.0 days, *P* < .001; Dacombe et al^16^: *P* = .0008), dialysis (OR 3.44, 99% CI 1.61-7.35; Lee et al^48^), chronic obstructive pulmonary disease (OR 1.49, 99% CI 1.19-1.86; Lee et al^48^), bleeding disorders (OR 2.58, 99% CI 1.65-4.02; Lee et al^48^), Parkinson disease (2.9 ± 2.5 vs. 2.1 ± 2.3 days; Papalia et al^69^), cirrhosis (2.6 ± 3.6 vs. 1.5 ± 1.1 days; Martino et al^58^), and metabolic syndrome (adjusted OR 1.13-1.44; Fassler et al^22^; Murphy et al^66^). Other predictors included functional dependence (OR 2.73, 99% CI 2.05-3.64; *P* < .001; Lee et al^47^), sepsis (OR 3.34, 99% CI 1.60-6.96; *P* < .001; Lee et al^47^), and dyspnea (OR 1.37, 99% CI 1.10-1.72; *P* < .001; Lee et al^47^), while smoking was not consistently significant (OR 1.20, 99% CI 0.97-1.48; *P* = .016 univariate, no significance in multivariance; Hartwell et al^31^).

## Physiological and laboratory markers

### Anemia/hemoglobin-related markers

Anemia was consistently associated with prolonged LOS across seven studies.^2^^,^^10^^,^^19^^,^^39^^,^^63^^,^^72^^,^^82^ Polisetty et al^72^ reported a mean LOS of 3.3 days in patients with iron-deficiency anemia vs. 2.4 days in non-anemic controls (*P* < .0001). Burns et al^10^ observed similar findings (5.0 ± 4.0 vs. 2.2 ± 1.6 days; *P* < .001), and Doan et al^19^ demonstrated increasing LOS with the degree of anemia: 1.5 days for normal hematocrit, 2.1 days for mild anemia, and 3.0 days for moderate-to-severe anemia (*P* < .0001). Large cohort analyses reinforced this relationship: Wang et al^82^ reported LOS >2 days in 1.4% of non-anemic patients, 2.9% of mild anemia (OR 1.67, 95% CI 1.21-2.30; *P* = .002), and 12.5% of moderate-to-severe anemia patients (OR 6.61, 95% CI 4.84-9.04; *P* < .001). Alder et al^2^ similarly observed mean LOS of 1.8 ± 1.3, 2.3 ± 2.0, and 3.0 ± 2.6 days for non-anemic, mild, and moderate-to-severe anemia, respectively, with anemia severity defined using WHO hemoglobin thresholds (*all P* < .001). Blood loss-related anemia independently predicted prolonged LOS, with Kandil et al^39^ reporting OR 1.65 (95% CI 1.21-2.23; *P* < .001) and Miltenberg et al^63^ reporting OR 1.43 (95% CI 1.21-1.69; *P* < .001). Kishan et al^44^ similarly identified pre-operative hematocrit <35% as strongly predictive of prolonged LOS in patients undergoing revision shoulder arthroplasty (OR 4.3, 95% CI 2.1-8.6).

### Malnutrition

Malnutrition similarly predicted prolonged LOS.^10^ Burns et al^10^ reported LOS of 3.7 ± 2.8 days in malnourished patients vs. 2.2 ± 1.5 days in well-nourished controls (*P* < .001). Using the Geriatric Nutritional Risk Index (GNRI), Liu et al^54^ demonstrated a dose-response relationship: prolonged LOS (>2 days) occurred in 13.0% of patients with normal nutrition (GNRI >98), 24.7% with moderate malnutrition (GNRI 92-98; OR 1.85, 95% CI 1.63-2.12; *P* < .001), and 41.6% with severe malnutrition (GNRI <92; OR 3.27, 95% CI 2.73-3.92; *P* < .001).

### Hypoalbuminemia

Hypoalbuminemia was independently associated with extended LOS in 3 studies.^23^^,^^26^^,^^48^ Lee et al reported mean admission-to-discharge LOS of 3.46 days in hypoalbuminemia patients vs. 2.26 days in matched controls (*P* < .001), with post-operative LOS similarly increased (2.75 vs. 2.20 days; *P* < .001).^47^ Garcia et al found hypoalbuminemia conferred 69% higher adjusted odds of prolonged LOS (OR 1.69, 95% CI 1.02-2.78; *P* = .041).^26^ Flamant et al demonstrated that low pre-operative albumin (<3.5 g/dL) predicted extended LOS (>3 days) in primary shoulder arthroplasty (26.8% vs. 13.6%; multivariable OR 2.11; *P* = .03), while in revision shoulder arthroplasty, the association was not significant.^23^

### Hyponatremia and alkaline phosphatase

Electrolyte disturbances were also predictive of increase in LOS in 3 studies.^47^^,^^49^^,^^53^ Hyponatremia (<135 mEq/L) was associated with increased LOS in both Lee et al^47^ (OR 1.63, 99% CI 1.31-2.03) and Liu et al^49^ (29.4% vs. 17.7% prolonged LOS; multivariate OR 1.63, 95% CI 1.09-2.45; *P* = .017). Elevated alkaline phosphatase was similarly associated with extended LOS (>2 days) in Liu et al^53^ (40.7% vs. 17.7% for normal alkaline phosphatase; OR 2.37, 95% CI 1.31-4.30; *P* = .004).

### Dehydration

Dehydration was independently associated with prolonged LOS in 2 studies.^43^^,^^50^ In both, dehydration was defined biochemically using the pre-operative blood urea nitrogen (BUN) to creatinine (Cr) ratio. Liu et al^50^ classified patients with BUN/Cr ≥ 25 as dehydrated, reporting LOS >2 days in 25.8% vs. 15.1% in normally hydrated patients (OR 1.50, 95% CI 1.05-2.14; *P* = .028). Kim et al^43^ further stratified severity by BUN/Cr into non-dehydrated (<20), moderately dehydrated (20-25), and severely dehydrated (>25), showing stepwise increases in LOS ≥2 days from 34.1% to 35.5% and 38.6%, respectively (*P* < .001), with similar trends observed in elderly sub-groups.

## Medications

### Opioid

Pre-operative opioid exposure demonstrated significant associations with post-operative hospital LOS in 7 studies.^8^^,^^10^^,^^45^^,^^60^^,^^62^^,^^84^ Opioid use was consistently linked with dose-dependent increases in LOS. Mayfield et al^60^ reported mean LOS increasing across opioid exposure quintiles from <22.3 morphine milligram equivalents (1.37 ± 1.72 days) to >83.3 morphine milligram equivalents (2.33 ± 2.24 days; *P* < .0001), while Brandner et al^8^ observed longer LOS among opioid-dependent patients compared with non-dependent patients (1.8 ± 1.8 vs. 1.6 ± 1.7 days; *P* < .001). In multivariate analysis, Wilson et al^84^ demonstrated a stepwise increase in odds of extended LOS (≥3 days) with escalating opioid use, with adjusted ORs of 1.25 for <1 oral morphine equivalents (OME)/d (*P* < .001), 1.29 for 1-5 OME/d (*P* = .002), 1.52 for 5-10 OME/d (*P* < .001), and 1.98 for >10 OME/d (*P* < .001), univariate extended LOS proportions similarly increased from 15.4% in opioid-naïve patients to 25.9% in patients receiving >10 OME/d. Kopriva et al^45^ corroborated these findings, reporting LOS >3 days in 15.5% of opioid-naïve patients, 19.2% with tramadol, 21.8% with traditional opioids, and 20.9% with combined opioids and tramadol (all *P* < .001), with adjusted ORs demonstrating a 31%-51% higher likelihood of extended LOS in opioid-exposed patients compared with opioid-naïve controls. Menendez et al^62^ further reported a higher prevalence of prolonged LOS among pre-operative opioid users (24% vs. 14%; *P* = .013). Burns et al^10^ found no significant difference in LOS between opioid-affected and unaffected patients (2.3 ± 1.7 vs. 2.2 ± 1.7 days; *P* = .407), highlighting variability across cohorts.

### Tranexamic acid

Tranexamic acid (TXA) demonstrated mixed effects on LOS in the two included studies.^3^^,^^61^ Mayfield et al^61^ reported no difference in propensity-matched cohorts (1.5 ± 1.0 days for both TXA and non-TXA patients; *P* = .629), although patients at high risk for venous thromboembolism experienced significantly shorter LOS with TXA (1.7 vs. 1.9 days; *P* = .0001). Anthony et al^3^ similarly observed reduced LOS among TXA users (1.8 ± 1.2 vs. 2.1 ± 1.9 days; *P* < .0001), corresponding to a 6.2% shorter adjusted LOS (95% CI −8.0% to −4.4%; *P* < .05).

### Steroid

Steroid administration demonstrated mixed effects on LOS in two studies.^7^^,^^31^ Biron et al^7^ found no significant association between perioperative steroid use and LOS (short LOS: 4.48%; long LOS: 5.77%), whereas Hartwell et al^31^ reported a higher proportion of patients with LOS >24 hours among steroid recipients (59.7% vs. 40.3%; *P* = .0059).

## Mental health conditions

Pre-operative mental health disorders were evaluated in seven studies, particularly depression, were consistently associated with prolonged hospital LOS following arthroplasty.^18^^,^^56^^,^^57^^,^^62^^,^^64^^,^^75^^,^^81^ Diamond et al^18^ reported a median LOS of 3 days in patients with a diagnosis of depression vs. 2 days in patients without a diagnosis of depression (*P* = .0007). Mollon et al^64^ similarly observed a longer mean LOS among depressed patients (2.5 ± 1.6 days vs. 2.2 ± 1.6 days; *P* < .001), while Lunati et al^57^ demonstrated that 22.9% of patients with pre-operative depression experienced extended LOS compared with 20.0% of non-depressed patients (*P* < .001), with depression independently predicting LOS ≥3 days (OR 1.23, 95% CI 1.12-1.36; *P* < .001). Regression analyses further confirmed this relationship, with Puzzitiello et al^75^ identifying depressive status as an independent predictor of longer LOS (β = 0.15, 95% CI 0.04-0.45; *P* = .022).^75^ Menendez et al^62^ reported that 39% of patients with depression had prolonged LOS compared with 21% of non-depressed patients (*P* < .001). Vanderham et al^81^ extended these findings to mental health disorders more broadly, showing that patients with pre-operative mental health diagnosis (depression, anxiety, or psychosis) were more likely to have LOS >2 days compared with those without mental health disorders (20% vs. 14%), with similar rates observed for anxiety (20%), depression (20%), and psychotic disorders (23%) (all *P* < .001). In contrast, Lonergan et al^56^ found no significant difference in LOS between patients with mood disorders and those without (1.3 vs. 1.1 days; *P* = .11).

## Discussion

### Principal findings

In this systematic review of 64 studies involving 2,435,166 patients, modifiable pre-operative factors associated with prolonged LOS following shoulder arthroplasty were identified across physiological, metabolic, and psychosocial domains. Anemia, malnutrition, hypoalbuminemia, diabetes, frailty, higher ASA grade, comorbidity burden, opioid exposure, and depression were consistently associated with longer admissions. In contrast, general obesity showed limited association, with only morbid obesity demonstrating a modest effect. These findings highlight physiological reserve, systemic disease optimization, and mental health as key targets for reducing LOS.

### Comparison with prior evidence

Berman et al^6^ (2019) performed the only previous systematic review on this topic, including 22 studies. They identified age >65 years, female sex, obesity (BMI ≥40 kg/m^2^), reverse arthroplasty, and lower hospital/surgeon volume as the main correlates of prolonged LOS and non-home discharge.^6^ Diabetes was noted but not examined in depth; laboratory markers, nutritional indices, opioid dose–response, dehydration, and mental-health diagnoses were not synthesized.^6^

The present review expands the evidence base to 64 studies (>2.4 million patients), restricts the scope exclusively to modifiable physiological, metabolic, and psychosocial predictors, incorporates the substantial post-2019 literature on these factors, and organizes the findings into clinically actionable domains. This shifts the contribution from a descriptive summary of risk factors to an integrated optimization framework that directly supports short-stay and same-day pathways.

### Clinical significance

The modifiable predictors identified offer clear targets for pre-operative optimization to reduce LOS and support short-stay shoulder arthroplasty. Anemia correction, nutritional supplementation (including protein and vitamin D), glycemic control, prehabilitation to improve functional reserve, structured opioid tapering, and mental health management have all been shown to enhance recovery, reduce complications, and shorten hospitalization.^1^^,^^9^^,^^13^^,^^14^^,^^68^^,^^71^^,^^77^ Integrating these interventions into an evidence-based pre-operative optimization bundle or enhanced recovery protocol addresses physiological, metabolic, and psychosocial factors simultaneously. Such structured pathways provide a practical framework to improve perioperative efficiency, facilitate early mobilization, and enable safe implementation of short-stay or same-day discharge models in contemporary shoulder arthroplasty.

### Key observations and mechanistic insights

Several modifiable pre-operative factors identified in this review are supported by meta-analyses showing clear impacts on post-operative outcomes. Hypoalbuminemia (<3.5 g/dL) prolongs hospital stay, with Llombart et al^55^ reporting a mean difference of 1.59 days (95% CI 0.86-2.32) in total shoulder arthroplasty patients, and Tischler et al^80^ showing higher odds of extended LOS (>3 days, OR 2.24), readmission (OR 1.88-2.92), reoperation (OR 2.20-3.60), and mortality (OR 3.93-14.57), likely reflecting impaired wound healing and systemic vulnerability, highlighting nutritional optimization as a modifiable strategy to improve outcomes. In 1.46 million total hip and knee arthroplasty patients, anemia increased LOS (+1.3-1.6 days), sepsis risk (OR 3.93), cardiopulmonary events (OR 2.4), and readmission (OR 1.58) (82), likely delaying recovery by impairing oxygen delivery, increasing transfusion requirements, and limiting mobilization.^24^^,^^46^^,^^85^

Psychosocial factors also affect LOS. Kremic et al^46^ found pre-operative depression increased LOS by 0.98 days (95% CI 0.35-1.62) and raised odds of extended LOS by 1.27 (95% CI 1.11-1.46) in over 27 million surgical patients. Depression may impair pain tolerance, reduce rehabilitation engagement, and trigger stress responses, which, combined with metabolic deficits, further delays recovery.^46^ These findings support a biopsychosocial optimization strategy incorporating mental health screening, nutritional and hematologic optimization, and functional prehabilitation to shorten LOS and improve recovery efficiency in shoulder arthroplasty.

### Limitations

All included studies were observational, with many at serious risk of bias due to confounding, missing data, and selective reporting, limiting causal inference. Pre-operative predictors, such as anemia, malnutrition, and hypoalbuminemia, may be highly confounded by baseline patient health, frailty, or comorbidity burden; therefore, our synthesis is exploratory rather than confirmatory. Substantial heterogeneity in arthroplasty type, LOS definitions, and perioperative protocols precluded meta-analysis. Most studies were US based, limiting generalizability. A further methodological consideration is the potential for overlapping patient populations across studies using the same large administrative databases. Exact double-counting cannot be quantified due to the absence of unique identifiers. However, studies differed in year ranges, inclusion criteria, and outcome definitions. Partial cohort overlap remains a limitation that may amplify database-specific biases. Finally, mental health data were inconsistently reported, potentially underestimating their impact.

### Future directions

Prospective, multicentre studies are needed to evaluate pre-operative optimization strategies targeting anemia, nutrition, glycemic control, opioid use, and mental health. These studies should adjust for baseline demographics, comorbidities, and medication use to identify independent predictors of LOS and determine the most effective protocols. Future work should also stratify analyses by arthroplasty type and indication to determine whether the identified predictors differ across these clinically distinct populations. Additionally, interventional trials testing structured pre-operative regimens could establish evidence-based pathways for short-stay or same-day shoulder arthroplasty across diverse health-care settings.

## Conclusion

Modifiable pre-operative physiological factors, comorbidity burden, and mental health are key predictors of hospital LOS after shoulder arthroplasty. Anemia, malnutrition, hypoalbuminemia, insulin-dependent or complicated diabetes, frailty, and depression consistently prolong LOS, while general obesity (BMI 30-40) has minimal impact. Targeted pre-operative optimization, including mental health assessment, can safely reduce LOS, improve efficiency, and support short-stay or same-day pathways, providing an evidence-based framework to enhance outcomes and resource utilization.

## Disclaimers

Funding: No funding was disclosed by the authors.

Conflicts of interest: The author, their immediate family, and any research foundation with which they are affiliated have not received any financial payments or other benefits from any commercial entity related to the subject of this article.
