# Supplementary material for: Modifiable pre-operative physiological predictors of length of stay following shoulder arthroplasty: a systematic review
Source: JSES Rev Rep Tech. 2026 Aug 24;6(4):100863. doi: 10.1016/j.xrrt.2026.100863 (PMC13633913; doi:10.1016/j.xrrt.2026.100863)
Supplement: Supplementary file 1 [file mmc1.docx]

Search String

(((length OR postoperative) AND Stay) OR "Length of Stay" OR "Postoperative Stay" OR LOS OR Hospital Stay) AND ((Shoulder OR "Total shoulder") AND (arthroplasty OR Replacement)) AND (preoperative predictors OR risk factors OR predictive factors OR Predict OR prognosis OR prognostic)

Supplementary table 1 – Search String
