# Supplementary material for: Modifiable pre-operative physiological predictors of length of stay following shoulder arthroplasty: a systematic review
Source: JSES Rev Rep Tech. 2026 Aug 24;6(4):100863. doi: 10.1016/j.xrrt.2026.100863 (PMC13633913; doi:10.1016/j.xrrt.2026.100863)
Supplement: Supplementary file 2 [file mmc2.docx]

| **PICO Element** | **Inclusion** | **Exclusion** |
| --- | --- | --- |
| **Population (P)** | • Adults undergoing total shoulder arthroplasty (anatomic or reverse)  • All sexes and BMI ranges  • Various indications: osteoarthritis, rotator cuff arthropathy, fractures, rheumatoid arthritis, revision procedures | • Paediatric or non-arthroplasty populations  • Animal studies |
| **Exposure / Intervention (I/E)** | • Patient-level predictors: demographics, comorbidities, lab markers, medications, mental health conditions | • Surgical technique only without patient factor analysis |
| **Comparison (C)** | • Reference groups within studies (e.g., non-obese vs obese, non-diabetic vs diabetic) | • No comparator or reference group |
| **Outcome (O)** | • Postoperative hospital length of stay (LOS) | • Outcomes unrelated to LOS |
| **Study Design** | • Retrospective or prospective cohort, case-control, cross-sectional  • English language | • Case reports, reviews, editorials, commentaries, conference abstracts only |

Supplementary table 2 – Inclusion and Exclusion Criteria
